# N-Terminal Coiled-Coil Structure of ATPase Subunits of 26S Proteasome Is Crucial for Proteasome Function

**DOI:** 10.1371/journal.pone.0134056

**Published:** 2015-07-24

**Authors:** Tomonao Inobe, Reiko Genmei

**Affiliations:** Frontier Research Core for Life Sciences, University of Toyama, 3190 Gofuku, Toyma-shi, Toyama, 930–8555, Japan; Universidad Autónoma de Madrid, SPAIN

## Abstract

The proteasome is an essential proteolytic machine in eukaryotic cells, where it removes damaged proteins and regulates many cellular activities by degrading ubiquitinated proteins. Its heterohexameric AAA+ ATPase Rpt subunits play a central role in proteasome activity by the engagement of substrate unfolding and translocation for degradation; however, its detailed mechanism remains poorly understood. In contrast to AAA+ ATPase domains, their N-terminal regions of Rpt subunits substantially differ from each other. Here, to investigate the requirements and roles of the N-terminal regions of six Rpt subunits derived from *Saccharomyces cerevisiae*, we performed systematic mutational analysis using conditional knockdown yeast strains for each Rpt subunit and bacterial heterologous expression system of the base subcomplex. We showed that the formation of the coiled-coil structure was the most important for the N-terminal region of Rpt subunits. The primary role of coiled-coil structure would be the maintenance of the ring structure with the defined order. However, the coiled-coil region would be also be involved in substrate recognition and an interaction between lid and base subcomplexes.

## Introduction

The ubiquitin-proteasome system (UPS) is one of the most important cellular protein degradation systems. In UPS, proteins to be degraded are first polyubiquitinated by a cascade of reaction of E1, E2, and E3 enzymes [1]. Subsequently, the polyubiquitinated proteins are delivered to the proteasome, where they are unfolded from their unstructured initiation sites and degraded into small peptides [2]. In addition to the housekeeping function of removing misfolded proteins from the cell, the proteasome degrades short-lived regulatory proteins that control many cellular functions. However, despite its crucial cellular functions of the proteasome as a center player of UPS, the detailed molecular mechanisms underlying protein degradation by the proteasome remain poorly understood.

The 26S proteasome consists of the 20S core particle (CP) and 19S regulatory particle (RP), which caps either end of CP. Subunits of CP form four heptameric rings, which stack on top of each other to form a cylindrical structure [3]. Because the proteolytic active sites are buried deep within the cylindrical structure, these are only accessible from the channel that runs along the long axis of the cylinder. The entrance of this channel is closed when CP is not capped by CP activators such as 19S RP. Even when the entrance is open beneath the RP cap, it is not wide enough for most folded proteins. Thus, proteins to be degraded by CP must be unfolded and translocated into the central cavity by capped RP. In fact, 19S RP is responsible for almost all steps before the final polypeptide digestion. This 19S RP can be further divided into the lid and base subcomplexes. The base subcomplex directly caps the entrance of CP, and the lid subcomplex further covers the top of the base—CP complex. Although the lid plays important roles in the recognition of polyubiquitinated proteins and removal of polyubiquitin chains, the base plays central key roles of the proteasome, such as substrate recognition, gate opening, and protein unfolding and translocation. Therefore, extensive research is required to elucidate the molecular mechanism of action of the base subcomplex.

**Fig 1:**
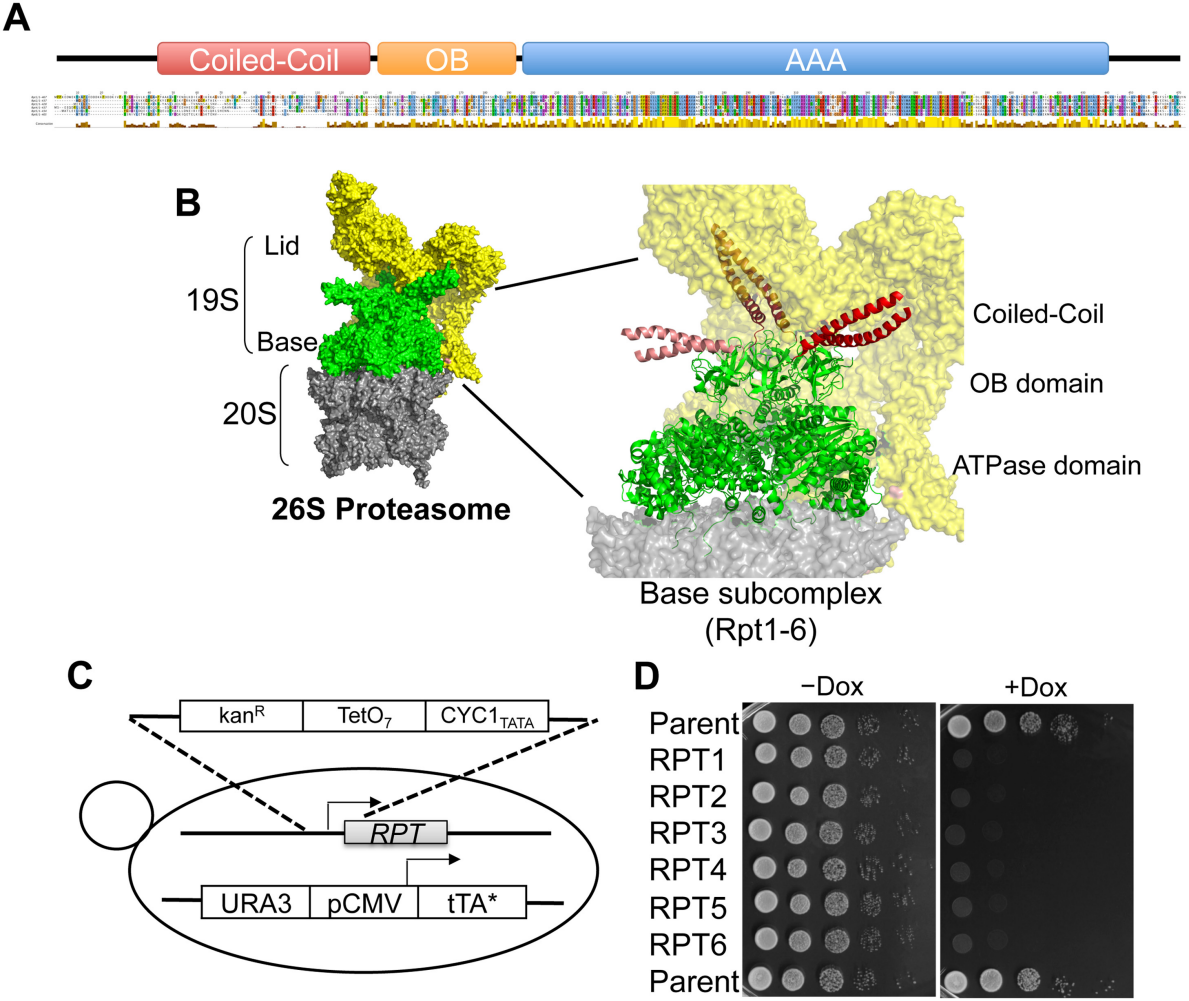
Structure of yeast Rpt subunits and construction of Rpt tet-off strains. (A) A schematic representation of structural domains of the Rpt subunits. Multiple sequence alignment of yeast Rpt subunits are indicated beneath the domain structure. Rpt1–6 were derived from *Saccharomyces cerevisiae*. Sequence conservation is indicated beneath the alignment, and conserved residues are marked and color-coded according to the default ClustalX settings. Enlarged sequence alignment is shown in S1 Fig. (B) Structure of 26S proteasome. Molecular surface of the 19S activator particle bound to the 20S core particle (CP; PDB ID 4B4T) (left). The 19S regulatory particle, which contains Rpt AAA+ ATPase subunits (green) and non-ATPase subunits (yellow), caps either end of the 20S CP (gray). Enlarged view of the Rpt AAA+ ATPase subunits are shown as a ribbon (right). N-terminal coiled coils formed by Rpt1–Rpt2 (light red), Rpt4–Rpt5 (red), and Rpt3–Rpt6 (dark red) are colored. Structures are produced by PyMOL. (C) Strain construction by one-step homologous replacement of native promoters with a TetO_7_-containing cassette. (D) Culture of Rpt tet-off strains was grown to early log phase (OD_600_ of approximately 0.6–0.8). Ten-fold serial dilutions of these cultures were spotted on YPDA medium agar plates or YPDA medium agar plates containing 10 μg/ml doxycycline. Plates were incubated at 30°C for 3 days and then photographed.

The base subcomplex mainly consists of a ring of six distinct AAA+ ATPase subunits designated Rpt1-6, which share highly homologous AAA+ ATPase domains (Fig 1a and S1 Fig). The molecular mechanism underlying substrate recognition and protein unfolding and translocation by the related bacterial homohexameric Clp proteases, ClpX, ClpA, and HslU, have been extensively studied [4]. Thus, it is possible to speculate on how the Rpt ring recognizes and unfolds substrate proteins. Loops that line the pore at the center of the ring of ATPase subunits grip the unstructured degron of the substrate protein and pull it, unfolding and translocating the substrate into the proteolytic active sites [5, 6]. However, the heterohexameric Rpt ring may employ a more or less different molecular mechanism from the homohexameric Clp proteases. Each Rpt subunit in unique heterohexameric proteasomal ATPase ring would have a distinct role in protein degradation, while ATPase subunit in homohexameric Clp proteases plays equivalent roles in substrate degradation.

Although the amino acid sequence of AAA+ ATPase domains of Rpt1–6 are highly homologous (Fig 1A and S1 Fig), ATPase activity and substrate-binding affinity of each Rpt subunit significantly differ from each other [7–12]. In contrast to the highly conserved AAA+ ATPase domains of Rpt1–6, the sequences of their N-terminal regions are poorly conserved (Fig 1A and S1 Fig). Thus, the heterologous N-terminal regions of Rpt subunits may contribute to the distinct function of each Rpt subunit. Recently, electron microscopy (EM) imaging of the structure of 19S RP revealed that the N-terminal regions of Rpt subunits form three distinct coiled-coil structures protruded from the hexameric ring (Fig 1B) [13–17], proposing the involvement of the N-terminal regions of Rpt subunits in the assembly of the Rpt ring and interaction with other proteins.

In this study, we investigated the requirements and roles of the N-terminal regions of six Rpt subunits derived from *Saccharomyces cerevisiae*. Systematic mutational analysis of the N-terminal regions of the Rpt subunits revealed that the conformation of the coiled-coil structure is the most important for their function. The coiled-coil conformation not only maintains the Rpt ring in a defined order but also may facilitate substrate recognition and maintain the interaction between the lid—base subcomplexes.

## Results

### Construction of Rpt tet-off strains

To investigate the roles of Rpt subunits in the proteasome, it is necessary to analyze the various properties of many mutant Rpt subunits. However, all Rpt subunits are lethal to almost all species, including yeast and higher species [8]. Therefore, it is difficult to analyze the function of mutant Rpt subunits that impair cell growth by disrupting proteasome function. Thus, we conducted rescue experiments using individual Rpt subunits in conditional knockdown strains for each Rpt subunit. This experiment allowed us to analyze the functional compensation of exogenic mutant Rpt subunits in cells in which the expression of endogenous Rpt subunits is conditionally suppressed.

Based on the method of Hughes *et al*, we constructed Rpt tet-off strains, which enabled us to conditionally knockdown individual *RPT* genes in the presence of doxycycline (Dox) [18, 19] (Fig 1C). A spotting growth assay for the constructed Rpt tet-off strains revealed that they were unable to grow on yeast peptone dextrose adenine (YPDA) medium agar plates containing 10 μg/ml Dox; however, they were able to grow on YPDA medium agar plate lacking Dox (Fig 1D). This indicates that gene silencing of Rpt subunits causes defective growth of yeast cells and confirms the lethality of Rpt subunits. Abnormal assembly of the Rpt ring was expected in cells in which the expression of Rpt subunits was suppressed. However, we did not observe such abnormal assembly of the Rpt ring in our experimental condition (S2 Fig). Probably a marginal disruption of the assembly of the Rpt ring lacking any one of the Rpt subunit is enough to cause the conditional severe growth defect in the presence of Dox.

### Mouse Rpt subunits can be substituted for yeast Rpt subunits

**Fig 2:**
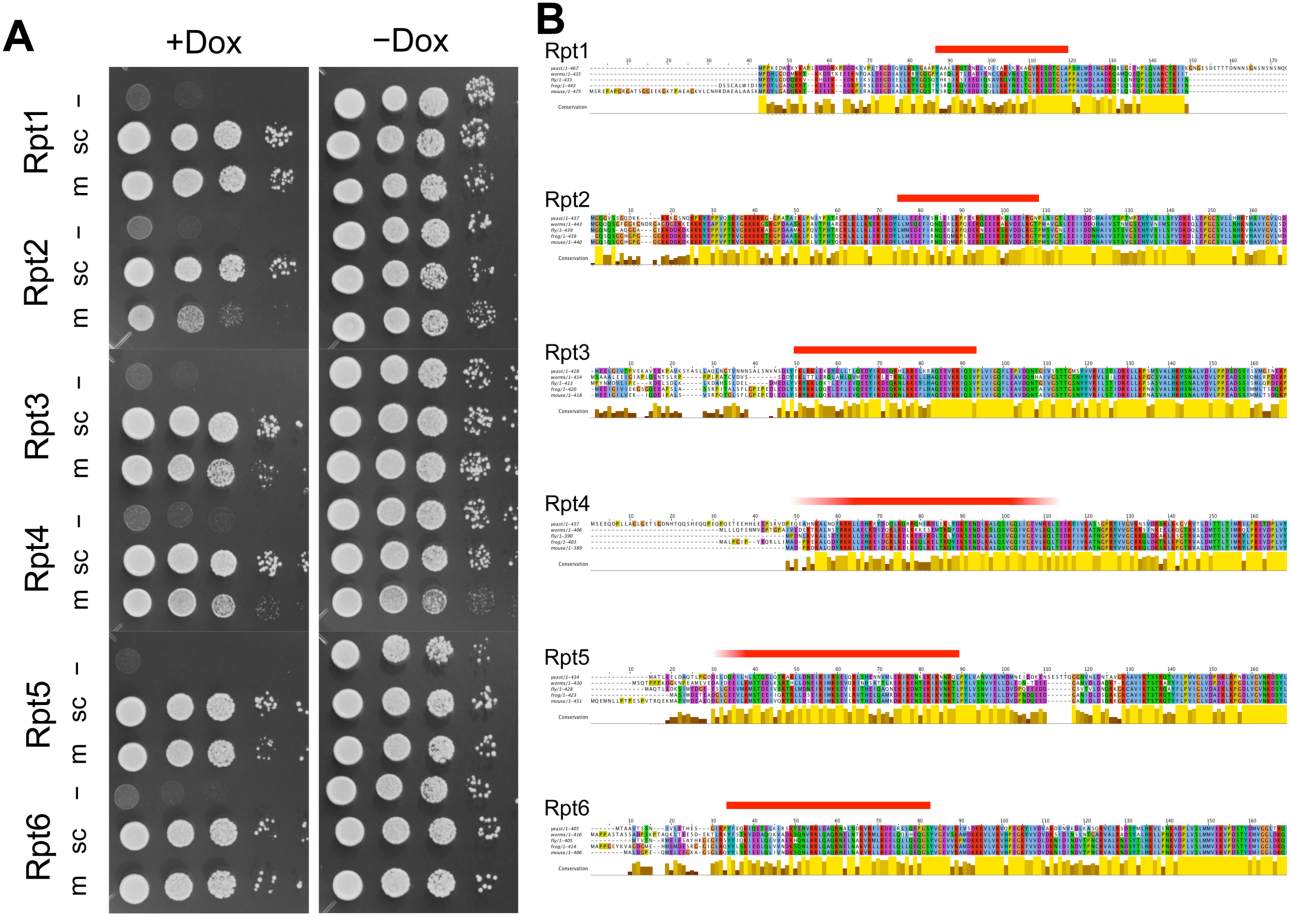
Expression of mouse and yeast Rpt subunits rescues the growth of yeast with conditional suppression of the wild-type yeast Rpt subunit. (A) Rpt tet-off strains were transformed with the pAUR123 yeast expression vector encoding yeast (sc) and mouse (m) Rpt subunits, grown to early log phase and individually spotted in duplicate as ten-fold serial dilutions on plates either without (right panel) or with (left panel) doxycycline. Plates were incubated at 30°C for 2 days and then photographed. (B) Multiple sequence alignment of the N-terminal regions of Rpt subunits derived from yeast, worm, fly, frog, and mouse. The coiled-coil regions of Rpt subunits predicted by PairCoil2 are indicated above the sequences (ref. S6 Fig). Sequence conservation is indicated beneath the alignment, and conserved residues are marked and color-coded according to the default ClustalX settings. Multiple sequence alignments of full-length Rpt subunits are shown in S3 Fig.

We investigated whether the expression of various exogenous Rpt mutants encoded in the pAUR123 vector was able to compensate for conditionally suppressed endogenous wild-type yeast Rpt subunits. The expression of N-terminally human influenza hemagglutinin (HA)-tagged exogenous Rpt subunits was low because the copy number of the CEN-type autonomous vector pAUR123 in yeast was suppressed and transcriptional activity of the ADH1 promoter, which regulates Rpt subunit expression, was weak. Although overexpression of some proteins was reported to induce cellular dysfunction, such as growth defect [20–22], it was not observed in all yeast cells expressing low level of exogenous Rpt subunits described here in the absence of Dox. The expression of the exogenous HA-Rpt subunits in yeast was detected using the anti-HA antibody during western blotting analysis of transformed yeast cells (S3, S7, and S8 Figs). A growth rescue assay with exogenous wild-type yeast Rpt subunits showed that the expression of each wild-type Rpt subunit could rescue yeast strains in which the respective Rpt gene was conditionally suppressed in the presence of Dox (Fig 2A). These data confirmed the validity of further rescue assays using mutant Rpt subunits in this assay system.

Following this, we examined whether mouse Rpt subunits could be substituted for the corresponding yeast Rpt subunit. Although the overall sequence homology between mouse and yeast Rpt subunits is relatively high, some regions are highly conserved, while others are not so conserved. Thus, this compensation experiment would give us a clue regarding the region that is critically important for each Rpt subunit. We found that five of six mouse Rpt subunits (Rpt1, Rpt3, Rpt4, Rpt5, and Rpt6) were able to rescue the suppression of the corresponding yeast Rpt subunit as well as yeast wild-type Rpt subunits did (Fig 2A). Mouse Rpt2 rescued the expression of yeast Rpt2; however, a weak growth defect persisted (Fig 2A). The expression of mouse Rpt subunits in yeast was confirmed by western blotting analysis (S3 Fig). This complementation was basically specific for the respective mouse orthologs (TI & RG, unpublished data).

Multiple alignments of the amino acid sequences of Rpt subunits derived from various species (yeast, warm, fly, frog, and mouse) indicated that the N-terminal 40–90 residues and C-terminal 10–30 residues or Rpt subunits are poorly conserved, whereas almost all other regions, including the AAA+ ATPase domains, are well conserved among all eukaryotes (Fig 2B and S4 Fig). Combining the experimental data regarding mouse Rpt orthologs and multiple alignments, we hypothesized that N- and C-terminal nonconserved regions do not have any functions. However, reportedly conserved C-terminal hydrophobic-tyrosine-X motifs are essential for the Rpt ring to associate with 20S CP and open its gated channel [23–26]. Thus, the nonconserved regions close to the N terminus of Rpt subunits may also play a genetically hidden but functionally important role in proteasome activity. The predicted secondary structure propensity of all Rpt subunits showed that N-terminal nonconserved regions have significantly low propensities for any secondary structures and that the coiled-coil structures are located at boundary regions between nonconserved and conserved regions (Fig 2B, S5 and S6 Figs). Thus, we speculated whether nonconserved N-terminal regions, including the predicted coiled-coil region, are dispensable for yeast growth.

### N-terminal coiled-coil regions of Rpt subunits are necessary for normal proteasome function

**Fig 3:**
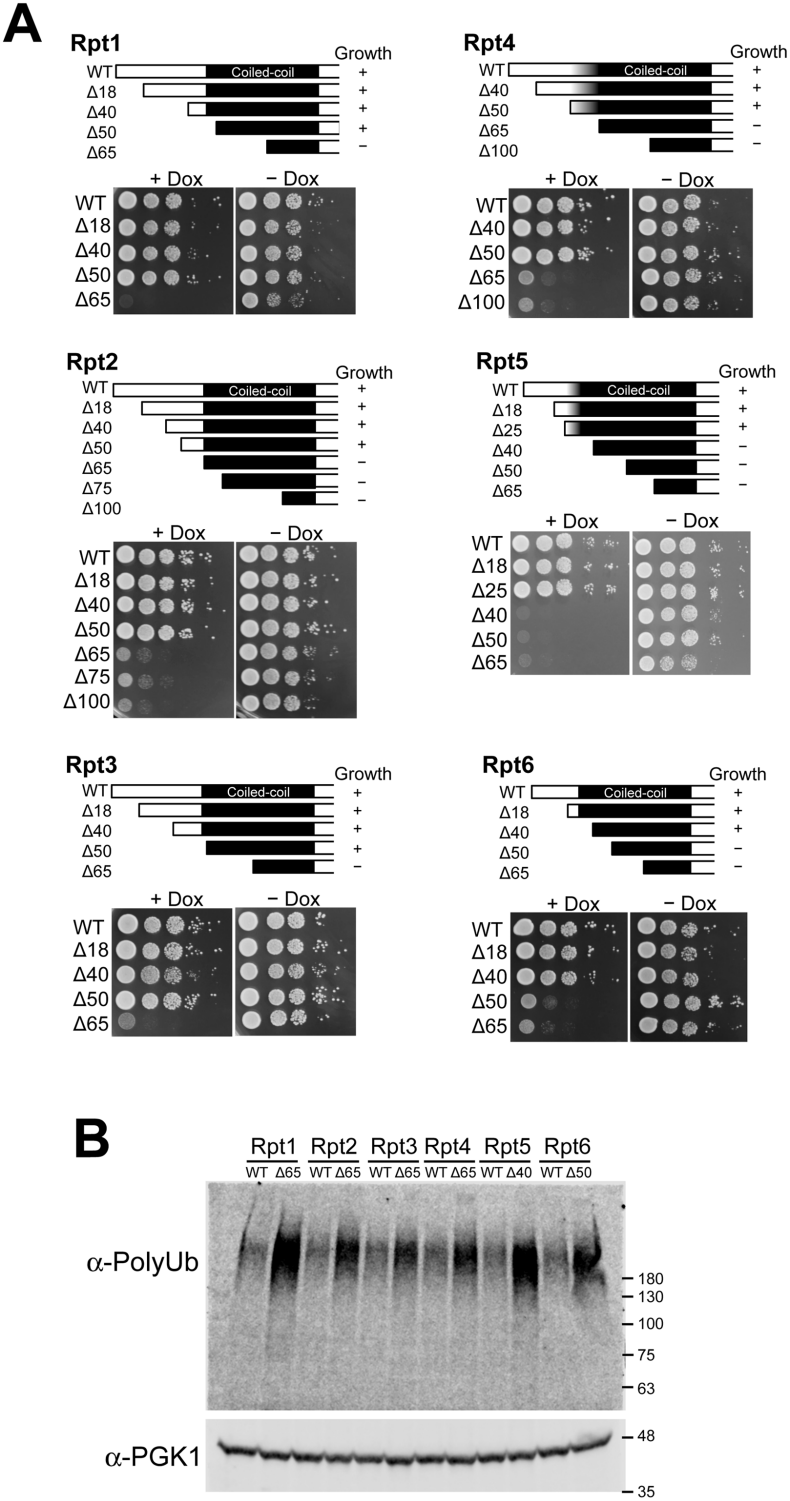
Coiled-coil region of Rpt subunits is required for proteasome function. (A) We constructed a series of deletion mutants (upper figures) in the pAUR123 vector and expressed them in the Rpt tet-off strains. Yeast cultures were grown to early log phase (OD_600_ of approximately 0.6–0.8). Ten-fold serial dilutions of these cultures were spotted on YPDA medium agar plates or on YPDA medium agar plates containing 10 μg/ml doxycycline (Dox). Plates were incubated at 30°C for 2 days and then photographed (lower panels). (B) Deletion of N-terminal coiled-coil region of Rpt subunits induces the accumulation of polyubiquitinated proteins. Accumulation of polyubiquitinated proteins in the Rpt tet-off yeast cells expressing wild-type and deletion mutants (Rpt1Δ65, Rpt2Δ65, Rpt3Δ65, Rpt4Δ65, Rpt5Δ40, and Rpt6Δ50) were analyzed using western blot with an anti-polyubiquitin antibody. After yeast cells at early log phase were treated with 20 μg/mL Dox for 3 h, cells were harvested and lysed with glass beads in the presence of 10% trichloroacetic acid (TCA) to preserve ubiquitination patterns. PGK1 was used as a loading control.

To investigate the roles of the N-terminal regions of Rpt subunits, we constructed a series of N-terminal deletion mutants of each yeast Rpt subunit. The expression of the deletion mutant series was confirmed in Rpt tet-off strains (S7 Fig). We then performed a rescue assay for the deletion mutant series of each yeast Rpt subunit (. 3). We found that deletion mutants of the N-terminus 20–50 residues of each Rpt subunit were still able to rescue the suppression of full-length Rpt subunits, although the maximum length that could be deleted while retaining growth differed between Rpt subunits (Fi. 3). For example, the N-terminal 50 residues of Rpt1, Rpt2, Rpt3, and Rpt4 could be deleted without causing defective yeast growth, whereas deletion of the N-terminal 40 amino acid of Rpt5 or 50 amino acid of Rpt6 was sufficient to impair yeast growth. Although the expression level of deletion mutants of each Rpt subunit differed from each other, we could not observe any clear correlations between the expression level of deletion mutants and their ability to rescue the knockdown of wild-type Rpt subunits (Fig 3 and S7 Fig). Because the N-terminal regions that could be deleted without causing defective growth are not highly conserved among species and are not predicted to form any rigid secondary structures, we concluded that these deletable regions do not play any role in proteasome function (Figs 2B and 3, S4 and S5 Figs). However, once the N-terminal deletions reached highly conserved regions predicted to form coiled-coil structure, the deletion mutants failed to rescue suppressed full-length Rpt subunits (Figs 2B and 3)(S6 Fig). Although the correlation between yeast viability and predicted coiled-coil region are not perfect (Fig 3), these results suggest that the N-terminal conserved coiled-coil regions are necessary for proteasome function.

Dysfunction of the proteasome is known to result in the accumulation of polyubiquitinated proteins in the cell. To test whether the conserved N-terminal coiled-coil regions are inevitable for proteasome function, we examined the amount of polyubiquitinated protein in the Rpt tet-off yeast cells expressing wild-type and deletion mutant of respective Rpt subunits, following the gene suppression of respective endogenous Rpt subunit by Dox using western blotting. All tet-off cells with deletion mutants, which were unable to compensate for the suppression of full-length Rpt subunit, showed more accumulated polyubiquitinated proteins than the cells with wild-type Rpt subunits at 3 h after adding Dox (Fig 3B). The extent of polyubiquitin accumulation in yeast cells with respective deletion mutant was different from each other. For example, Rpt1Δ65 and Rpt5Δ40, which showed the most severe growth defect, exhibited the most accumulated polyubiquitinated proteins among six deletion mutants. These results clearly indicate that the N-terminal conserved coiled-coil of each Rpt subunit plays an important role in proteasomal protein degradation probably with a different contribution.

### Destabilization of the N-terminal coiled-coil regions of Rpt subunits causes proteasome defects

**Fig 4:**
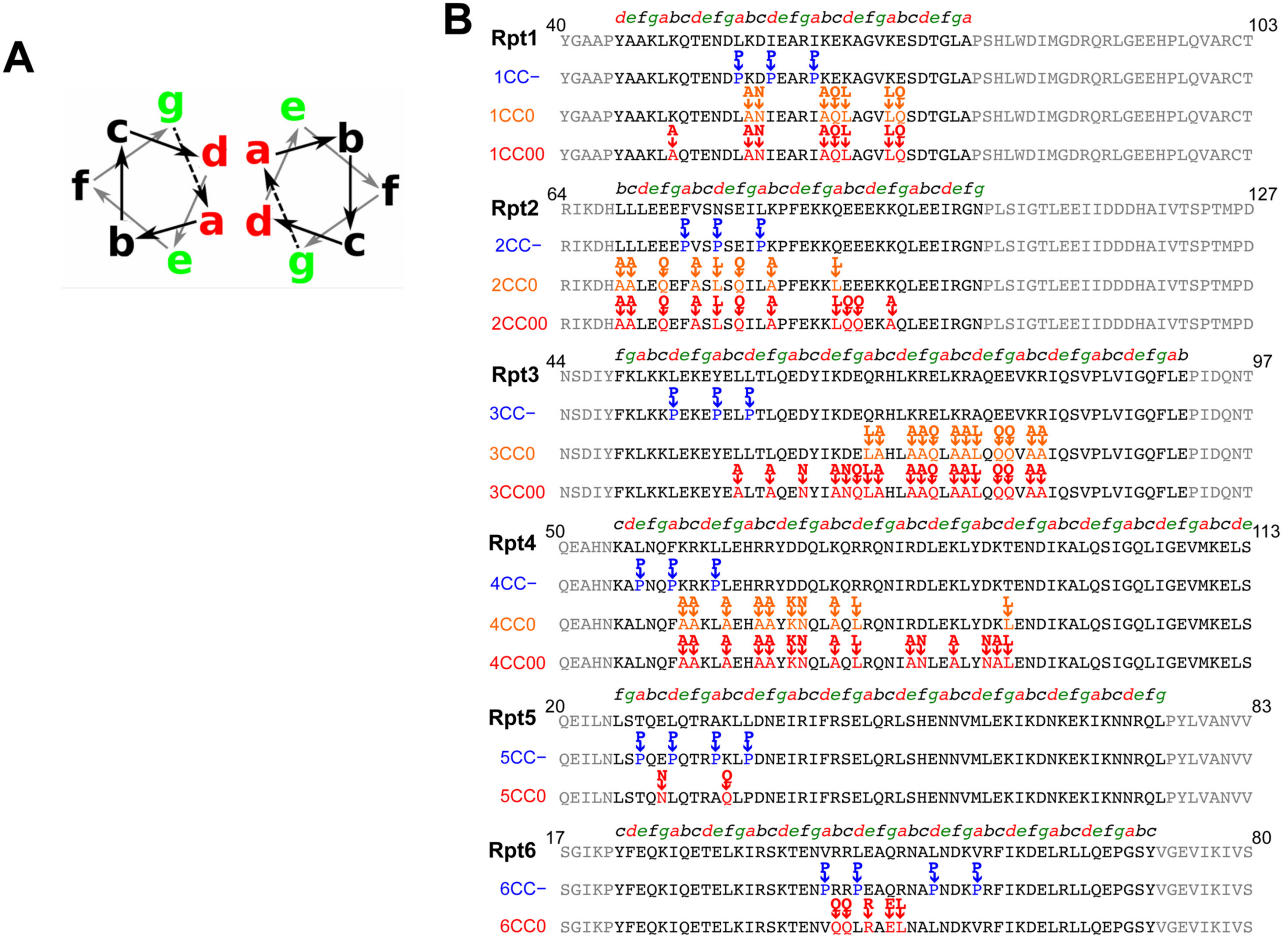
Structure-guided mutagenesis of Rpt subunits. (A) Schematic zenithal representation of two coiled alpha helices. Red, heptad positions a/d; green, g/e; and black, b, c, and f. (B) Coiled-coil-destabilizing (CC−) and coiled-coil-neutral (CC0 and CC00) mutants are grouped as Rpt1–6 subunits. Partial sequences are shown as follows: Rpt1 (40–103); Rpt2 (64–127); Rpt3 (44–107); Rpt4 (50–113); Rpt5 (50–83); and Rpt6 (17–80). The positions of the coiled-coil heptad repeat (abcdefg) are the indicated above sequences.

**Fig 5:**
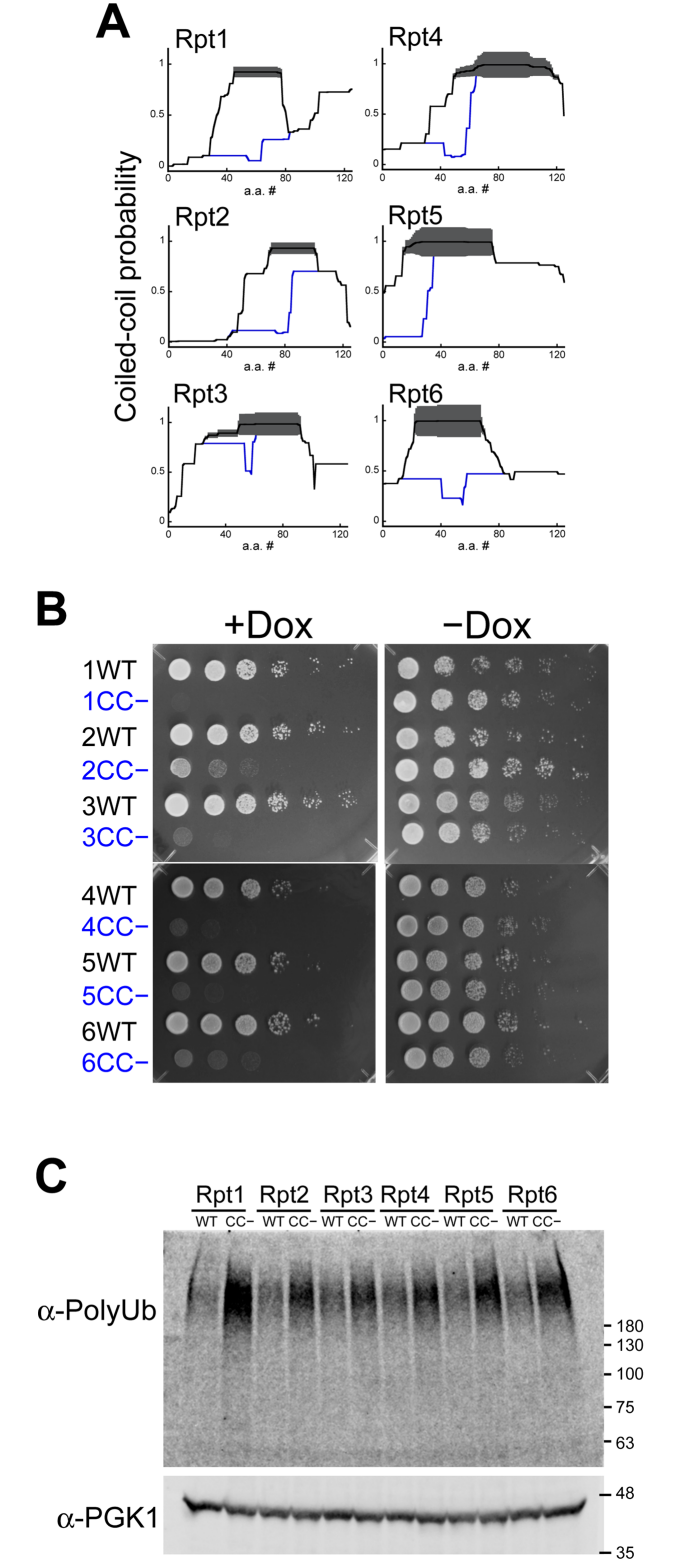
Coiled-coil mutations reveal that destabilization of Rpt subunits hampers yeast growth. (A) Coiled-coil probability for wild-type (black lines) and CC− mutants (blue lines) of Rpt subunits calculated by PairCoil2. The thickness of the red lines represents the confidence of the prediction (p-scores) by Paircoil2. (B) Rpt tet-off strains were transformed with the pAUR123 yeast expression vector encoding wild-type and CC− mutants of Rpt subunits, grown to early log phase, and individually spotted in duplicate as a ten-fold serial dilution on plates either without (right panel) or with (left panel) doxycycline. Plates were incubated at 30°C for 2 days and then photographed. (C) Disruption of the coiled-coil conformation of Rpt subunit induces the accumulation of polyubiquitinated proteins. The accumulation of polyubiquitinated proteins in the Rpt tet-off yeast cells expressing wild-type and CC− mutants (Rpt1CC−, Rpt2CC−, Rpt3CC−, Rpt4CC−, Rpt5CC−, and Rpt6CC−) were analyzed using western blot with an anti-polyubiquitin antibody. After yeast cells at early log phase were treated with 20 μg/mL Dox for 3 h, cells were harvested and lysed with glass beads in the presence of 10% TCA to preserve ubiquitination patterns. PGK1 was used as a loading control.

To further define the importance of the N-terminal coiled-coil regions of yeast Rpt subunits, we investigated whether mutations in these regions were able to rescue the knockdown of wild-type Rpt subunits. Coiled coils contain repeats of seven residues in which a hydrophobic amino acids occupy positions a/d, where two coiling helices bind to each other (Fig 4A). In contrast, hydrophilic polar or charged residues are exposed to the outer surface, and charged residues at positions e/g can form coiled-coil-stabilizing salt bridges [27] (Fig 4A). These heptad repeats can be observed in the N-terminal coiled-coil regions of Rpt subunits (Fig 4B). Thus, we used structure-guided mutagenesis to modulate the stability of the putative coiled-coil region. First, coiled-coil-defective (CC−) mutants were constructed by substituting a couple of residues at the a/d positions with proline which is commonly used as a structural disruptor (Fig 4B). CC− mutations were predicted to significantly reduce the coiled-coil propensity (Fig 5A). To determine the effect of coiled-coil destabilization in yeast, we performed the rescue assay with CC− Rpt subunits. We found that all CC− mutants were unable to compensate for the suppression of wild-type Rpt subunits and facilitate yeast growth (Fig 5B). Western blot analysis revealed that the expression levels of CC− Rpt subunits in yeast cell were the same level as those of wild-type Rpt subunits (S8 Fig). However, Rpt3CC−, which is predicted to have relatively small destabilization of the coiled-coil region, showed strong growth defect, whereas Rpt6CC−, which is predicted to have completely abolished coiled-coil propensity, showed a weak rescue of knockdown in early dilution (Fig 5B). These contradictory observations may reflect the following contributions of each coiled-coil region of Rpt subunits: contributions to coiled-coil integrity, substrate recognition, and lid—base interaction. It is also possible that coiled-coil prediction software, which is designed to predict the location of coiled-coil region, may not correctly predict coiled-coil integrity. Furthermore, to test whether the coiled-coil conformation is required for proteasome function, the amount of polyubiquitinated protein in the Rpt tet-off yeast cells with CC− mutants were compared with those expressing corresponding wild-type Rpt subunits after the addition of Dox. Polyubiquitinated proteins were more accumulated in the cells expressing CC− mutants (Fig 5C). Among six CC− mutants, Rpt1CC−, which showed the most severe growth defect, showed the most accumulated polyubiquitinated proteins. These results suggest that the conformation of the N-terminal coiled-coil is required for the normal function of the proteasome.

### Base subcomplex formation by mutant Rpt subunits

**Fig 6:**
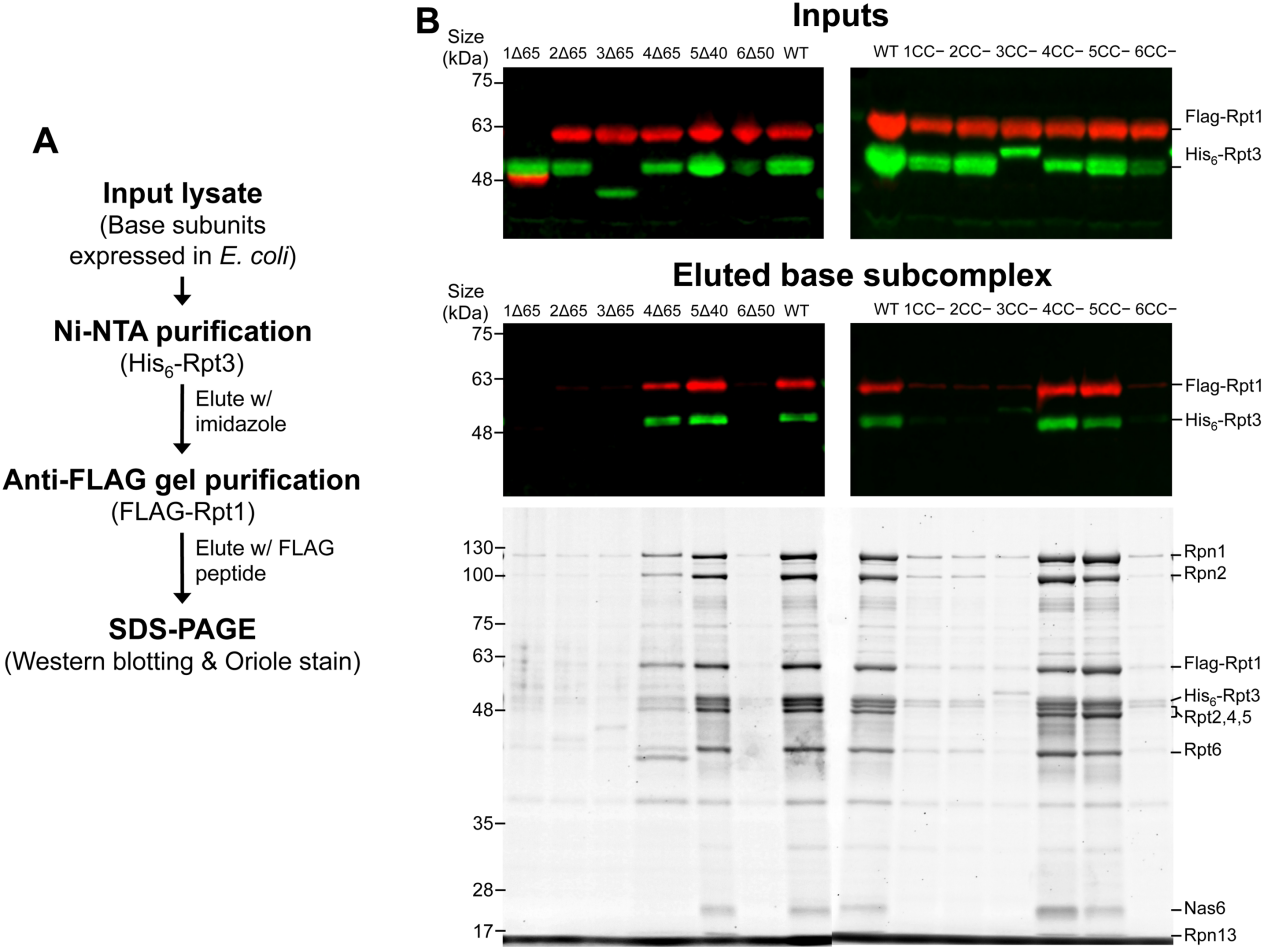
Base subcomplex formation by mutant Rpt subunits. (A) Schematic representation of the TAP procedure used to isolate the assembled base subcomplex from *E*. *coli* lysate. (B) Total lysates (top panels) and eluted fraction (middle and bottom panels) were separated by sodium dodecyl sulfate polyacrylamide gel electrophoresis (SDS-PAGE). (Top and middle panels) Western blotting analysis: FLAG-tagged Rpt1 (red) and His_6_-tagged Rpt3 (green) were revealed with anti-FLAG and anti-His_6_ antibodies, respectively. (Bottom panels) The eluted fraction was separated by SDS-PAGE and visualized using Oriole staining. Deletion mutants (Rpt1Δ65, Rpt2Δ65, Rpt3Δ65, Rpt4Δ65, Rpt5Δ40, and Rpt6Δ50; left panels) and coiled-coil destabilizing (CC−) mutants (Rpt1CC−, Rpt2CC−, Rpt3CC−, Rpt4CC−, Rpt5CC−, and Rpt6CC−; right panels) that caused the defective growth of yeast cells were analyzed as above. In the input of (B), FLAG-Rpt1Δ65 appeared to have lower molecular weight due to its N-terminal deletion because FLAG-tag was attached to Rpt1. With the similar reason, His_6_-Rpt3Δ65 shows smaller molecular weight. In contrast, His_6_-Rpt3CC− appeared slightly higher in position than wild-type His_6_-Rpt3 probably because CC− mutation (Pro replacement) lowered the migration of His_6_-Rpt3CC−. For the same reason, HA-Rpt3CC− also appeared slightly higher in position than wild-type HA-Rpt3 (S8 Fig).

Because constructed mutants that induce defective yeast growth tend to destabilize the coiled-coil structure, the abnormal assembly of the hexameric ring structure of Rpt subunits is assumed to occur in yeast cells that express coiled-coil mutants of Rpt subunits. However, it is difficult to investigate the assembly status of Rpt subunits in yeast cells because of the severe growth defect of cells expressing mutant Rpt subunits (ref. S2 Fig). Therefore, using the base subcomplex reconstitution system developed by Beckwith *et al* in *E*. *coli* [12], we investigated whether coiled-coil mutants of Rpt subunits formed the base subcomplex. In the base subcomplex reconstitution system, six Rpt subunits, Rpn1, Rpn2, and molecular chaperones for the assembly of the base subcomplex (Nas2, Nas6, Hsm3, and Rpn14), are co-expressed in *E*. *coli*. Among them, tetrahistidine (His_6_) and FLAG tags were attached to the N-termini of Rpt3 and Rpt1, respectively. Although the base subcomplex also comprises Rpn10, Rpn10 was omitted in this experiment because it was not co-purified with the isolated base subcomplex [12]. Therefore, the base subcomplex that successfully reconstituted in *E*. *coli* can be easily isolated by tandem affinity purification (TAP) using Ni-NTA agarose gel and anti-FLAG beads (Fig 6A). After the TAP of coexpressed wild-type base components, western blotting analysis was used to detect both FLAG-Rpt1 and His_6_-Rpt3, and the Oriole stain for all proteins was used to detect all subunits constituting the base subcomplex, indicating its successful reconstitution of base subcomplex (Fig 6B). Once the entire hexametric Rpt ring was formed, Nas2 and Hsm3 were released. Nas6 and Rpn14 (probably one of the bands between Rpt2, 4, 5 and Rpt6) remain bound to Rpt3 and Rpt6 until the holo 19S RP is formed [28]. In contrast to wild-type Rpt subunits, several Rpt mutants (Rpt1Δ65, Rpt2Δ65, Rpt3Δ65, Rpt6Δ50, Rpt1CC−, Rpt2CC−, Rpt3CC−, and Rpt6CC−), which caused defective growth in yeast cells, revealed only faint staining for base subunits, including FLAG-Rpt1 and His_6_-Rpt3, after TAP, indicating the defective assembly of the base subcomplex (Fig 6B). However, several other mutants of Rpt4 and Rpt5 (Rpt4Δ65, Rpt5Δ40, Rpt4CC−, and Rpt5CC−) successfully demonstrated the assembly of the base subcomplex (Fig 6B). These results indicate that the coiled-coil conformation of several Rpt subunits (Rpt1, Rpt2, Rpt3, and Rpt6) is critically important for the correct assembly of the base subcomplex. Furthermore, these results suggest that the coiled-coil conformation of two Rpt subunits (Rpt4 and Rpt5) may have additional functions, such as in substrate recognition.

### Coiled-coil mutations without destabilization of coiled-coils

In many cases, coiled-coil regions play important roles in protein—protein interaction, e.g., during oligomer formation and target recognition. The high-resolution EM structures of 19S RP suggest that the N-terminal coiled-coil protrusions are involved in the ring formation of Rpt subunits, their interaction with the lid subcomplex, and substrate recognition [13–17](Fig 1B). Thus, this requirement for the coiled-coil region along with above results suggest that coiled-coil regions are involved in substrate recognition or lid—base assembly as well as Rpt ring formation. Therefore, we examined how the coiled-coil domains of Rpt subunits maintain proteasome function by introducing further mutations into these regions.

**Fig 7:**
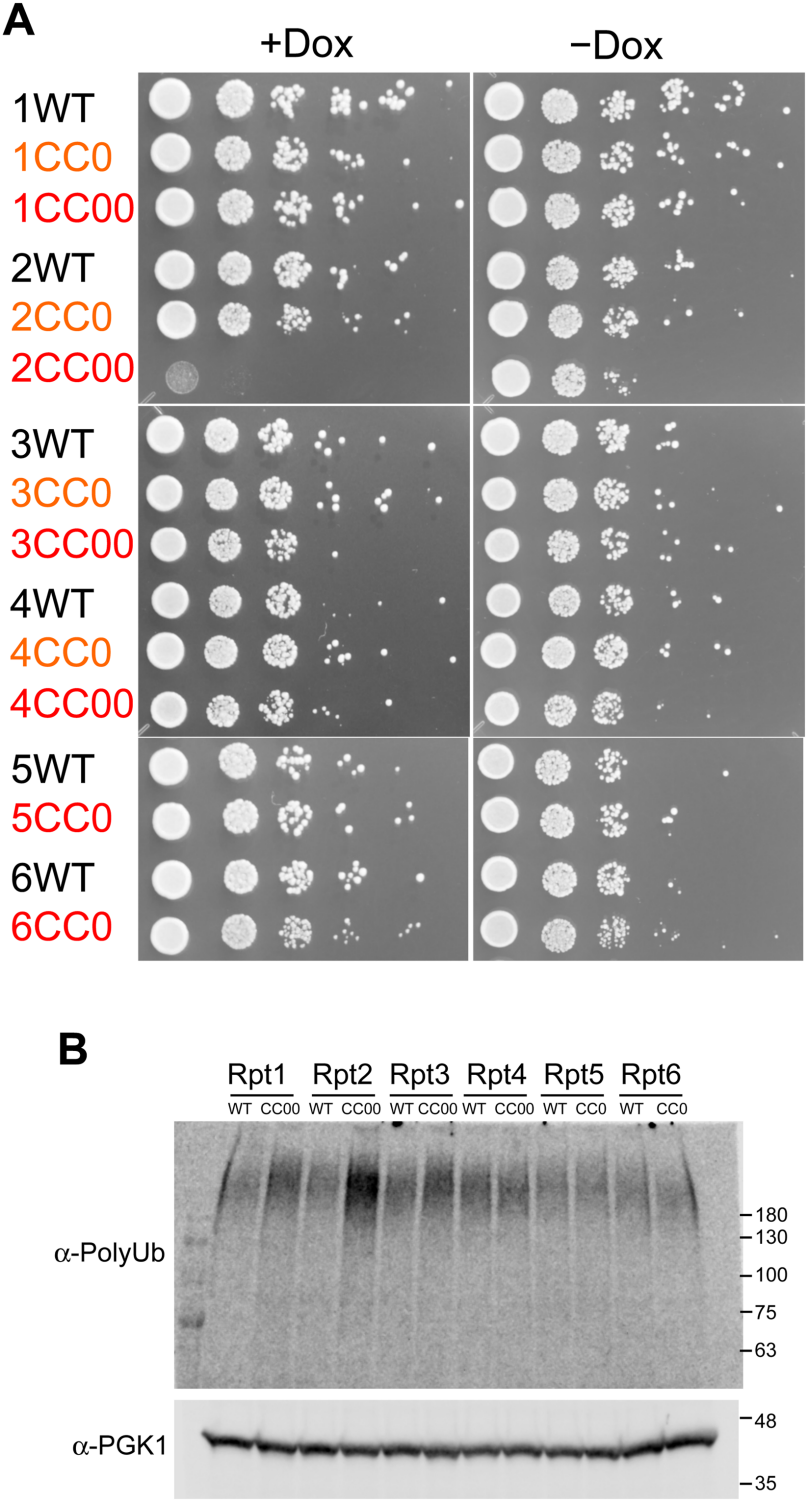
Coiled-coil mutations without destabilization of coiled-coils. (A) Rpt tet-off strains were transformed with the pAUR123 yeast expression vector encoding wild-type and CC0 and CC00 mutants of Rpt subunits, grown to early log phase, and individually spotted in duplicate as a ten-fold serial dilutions on plates either without (right panel) or with (left panel) doxycycline. Plates were incubated at 30°C for 2 days and then photographed. (B) Accumulation of polyubiquitinated proteins in the Rpt tet-off yeast cells expressing wild-type and CC0 or CC00 mutants (Rpt1CC00, Rpt2CC00, Rpt3CC00, Rpt4CC00, Rpt5CC0, and Rpt6CC0) were analyzed using western blot with an anti-polyubiquitin antibody. After the yeast cell at early log phase were treated with 20 μg/mL Dox for 3 h, cells were harvested and lysed with glass beads in the presence of 10% TCA. PGK1 was used as a loading control.

In contrast to the CC− mutant, mutation of residues exposed to the outside of the coiled-coil region (CC0 and CC00 mutation) cannot expected to destabilize coiled-coil conformation but may affect protein—protein interactions, such as those involving lid—base assembly and substrate recognition (Fig 4B). To keep the overall coiled-coil propensity constant, we constructed CC0 and CC00 mutants containing minimum numbers of mutations that enhance coiled-coil propensity. CC00 mutants contain more mutations in exposed residues than CC0 mutant. Because prediction software showed that coiled-coiled regions of wild-type Rpt5 and Rpt6 were intrinsically maximally optimized to form coiled-coil, it is very difficult to introduce many mutations without disturbing coiled-coil propensity. Thus, we prepared only CC0 mutants for Rpt5 and Rpt6. Although the number of mutational sites exceeded 10 (18 mutations at maximum), all CC00 mutants were still predicted to exhibit a coiled-coil propensity similar to wild-type coiled coils (S9 Fig). Rescue assays with CC0 and CC00 mutants showed that most CC0 and CC00 mutants succeeded in rescuing the suppression of wild-type Rpt subunits: only one CC00 mutant (Rpt2 CC00) abolished yeast growth in knockdown conditions (Fig 7A). The expression of these CC0 and CC00 mutants in yeast was confirmed by western blot analysis (S8 Fig). In the cell expressing most of the CC00 and CC0 mutants, except Rpt2 CC00, polyubiquitinated proteins were not accumulated upon the addition of Dox compared with the accumulation of polyubiquitinated proteins in cells with wild-type Rpt subunits, indicating normal proteasome function in the cell expressing CC0 and CC00 mutants of Rpt subunits (Fig 7B). Only Rpt2 CC00 showed an increased polyubiquitinated proteins, which would be caused by the disruption of the proteasome function (Fig 7B). Slightly increased polyubiquitinated proteins in the cells expressing Rpt1CC00 and Rpt3 CC00 may represent weak functional disruption of the proteasome, which did not cause a growth defect of the expressing yeast cells. These results indicate that the proteasome function is preserved while the coiled-coil propensity of each Rpt subunit, other than Rpt2, is kept high enough. Because mutations on residues exposed to the outside of the coiled-coil region of Rpt subunits (except Rpt2) showed no functional dysfunction of the proteasome, it is assumed that coiled-coil structure, rather than specific residues, of Rpt subunits other than Rpt2 may be important for substrate recognition or base—lid assembly as well as Rpt ring assembly. However, CC00 mutant of Rpt2 indicates that specific residue on the coiled-coil of Rpt2 may be involved in substrate recognition or base—lid assembly. For the substrate recognition and base—lid assembly, we speculated that both helical coiled-coil structure and specific residues exposed outside of the coiled-coil would be important, and that it would depend on each Rpt subunit. However, it was noted that the specific residues for substrate recognition would not be positioned in the outside for recognizing substrate without the backbone of coiled-coil structure (see Discussion).

## Discussion

In the present study, we found that the coiled-coil structure in the N-terminal regions of Rpt subunits is essential for proteasome function. In particular, the conformation of the coiled-coil region between adjoining N-terminal regions of Rpt subunits creates the structure of the Rpt ring, which is the ATPase engine of the proteasome. However, the coiled-coil region may also contribute to the lid—base assembly and substrate recognition in yeast. Here we discuss the role of the coiled-coil region of Rpt subunits in the proteasome.

The primary role of the coiled-coil region of Rpt subunits must be the assembly of the hexameric ring of Rpt subunits. Our results clearly showed that many Rpt mutants, wherein coiled-coil formation was disturbed, failed to form hexameric ring. Because, in combination with 19S RP, the hexameric Rpt ring is the core structure of the base subcomplex, defective hexameric ring formation by Rpt subunits as a result of coiled-coil destabilizing mutations would lead to the assembly defect of the 19S RP and 26S proteasome. Such assembly defect is assumed to result in defective growth. However, the coiled-coil destabilization of two Rpt subunits (Rpt4 and Rpt5) did not disturb the assembly of the base subcomplex; the destabilization of the coiled-coil region of these two Rpt subunits only disturbs the N-terminal half of the coiled-coil domain and may be insufficient to completely abolish the coiled-coil structure (Fig 5A). Further destabilization of the coiled-coil region will completely inhibit the correct assembly of the base subcomplex.

The sequences of the N-terminal coiled-coil-forming regions are highly diverse among six yeast Rpt subunits. One plausible reason for this may be the arrangement of each Rpt subunit to a specific position in the Rpt ring, evident in the specific coiled-coil conformations of Rpt1–Rpt2, Rpt4–Rpt5, and Rpt3–Rpt6 [29]. This specific order of Rpt subunits would provide a different ATPase activity and substrate-binding affinity of each Rpt subunit, resulting in the specialized role of each Rpt subunit during protein degradation [7–12]. Furthermore, our results along with the recent investigation of the three-dimensional structures of 19S RP suggested that the coiled-coils of Rpt subunits are involved in lid—base interaction and substrate recognition, in addition to Rpt ring formation [13–17].

Coiled-coil protrusions in the Rpt4–Rpt5 and Rpt1–Rpt2 pairs would play an important role in substrate recognition because they are exposed to the outside next to the central pore of the Rpt ring (Fig 1B). The mutations of Rpt4 and Rpt5 (Rpt4Δ65, Rpt5Δ40, Rpt4CC−, and Rpt5CC−), which do not disturb the assembly of the base subcomplex, have defective proteasome function and yeast growth (Figs 5 and 6). Because these mutations in Rpt4 and Rpt5 disturb the N-terminal region of the coiled-coil domain, this region must be involved in substrate recognition. Furthermore, the coiled-coil region of Rpt2 may participate in substrate recognition because the CC00 mutant of Rpt2 was unable to rescue the conditional knockdown of wild-type Rpt2 (Fig 7). The Rpt1 subunit, which forms a coiled-coil pair with Rpt2, is reportedly methylated at its N terminus [30]. The methylation of the N-terminal region of Rpt1 decreases cell growth and increases sensitivity to stress. Given that the Rpt1–Rpt2 coiled-coil region is exposed to the outside, this coiled-coil pair as well as the coiled-coil pair formed by Rpt4–Rpt5, may function in substrate recognition in yeast. Because the N-terminal regions of Rpt1, Rpt2, Rpt4, and Rpt5 are highly charged, the coiled-coil protrusions formed by Rpt1–Rpt2 and Rpt4–Rpt5 may recognize substrates by electrostatic interaction. After recognition, substrates will be transferred into the central pore of the Rpt ring and degraded into small peptides. The degradation of some substrates recognized by the N-terminal region of Rpt1-Rpt2 and Rpt4-Rpt5 subunits should be critically important for cellular activity. Even though proteins bound to the N-terminal region of these Rpt subunits would not be degraded by the proteasome, the protein binding to these Rpt subunits may induce the stimulation of ATPase activity of Rpt subunits [31–33]. Because ATPase activity is involved in many functions of the proteasome, such as assembly, protein unfolding, protein translocation, and deubiquitination, the induced ATPase-stimulation by bound proteins would also be important for such functions.

Rpt2 may play special roles in proteasome functions. We observed that mouse Rpt2 and CC00 mutant of Rpt2 were unable to rescue the knockdown of yeast wild-type Rpt2, while other mouse Rpt subunit and CC0 or CC00 mutants of other Rpt subunits were able to. It is also reported that Rpt2s derived from *Arabidopsis* and *Trypanosoma brucei* were unable to complement yeast mutants lacking Rpt2, whereas other five Rpt subunits derived from such species were able to complement the corresponding Rpt deletion mutant of yeast [34, 35]. Among equivalent mutants in ATP-binding region of six Rpt subunits, only Rpt2 mutant showed reduced peptidase activity of 20S CP [8, 10]. Moreover, Rpt2 played an important role in the assembly process of hexametric Rpt ring [23, 36]. Such special requirement and unique role of yeast Rpt2 would result in the growth defect of yeast cell expressing various Rpt2 mutants.

In contrast to coiled-coil region of Rpt1–Rpt2 and Rpt4–Rpt5, the coiled-coil protrusion in the Rpt3–Rpt6 pair may contribute to lid—base assembly rather than substrate recognition because it is buried in the lid—base interface region as shown in the recent EM structures (Fig 1B). Actually, the N-terminal regions of Rpt3 and Rpt6 have higher hydrophobicity than those of Rpt1, Rpt2, Rpt4, and Rpt5, which is more advantageous to keep the strong interaction between the lid and base subcomplexes. Here, we did not find any mutants that would affect only the interaction between the base and lid subcomplexes without destabilizing the base ring assembly. Once we find mutants that seem to be involved in the lid—base assembly, we will experimentally investigate the lid—base interaction in our future study.

As we suggested for the substrate recognition of Rpt1, Rpt2, Rpt4, Rpt5, we assume that lid-base assembly would also require both coiled-coil structure and outer exposed specific residues on the coiled-coil. Thus, we expected that the disruption of the outer-surface area of the coiled-coil of Rpt3 and Rpt6 by CC0 and CC00 mutations would affect lid—base interaction. However, CC0 and CC00 mutants of Rpt3 and Rpt6 showed no effect on yeast growth (Fig 7). Only Rpt2 CC00 showed proteasome dysfunction. Because at least two Rpt subunits forming a single coiled-coil and probably other subunits may cooperatively contribute to lid—base assembly as well as substrate recognition, mutations in a single Rpt subunit described here may be insufficient to disrupt such functions. Synthetic mutations of multiple subunits may disturb proteasome function. To clearly elucidate the contribution of N-terminal coiled-coil region of each Rpt subunit to the lid—base assembly and substrate recognition, we need to directly investigate the lid—base assembly and substrate recognition by introducing comprehensive mutations into multiple Rpt subunits.

## Materials and Methods

### Rpt tet-off yeast strains

For the tet-off system, yeast strains R1158 (URA3::CMV-tTA MATa his3-1 leu2-0 met15-0), Rpt2 tet-off (R1158 p*RPT2*::kan^R^-tetO_7_-TATA), Rpt4 tet-off (R1158 p*RPT4*::kan^R^-tetO_7_-TATA), and Rpt6 tet-off (R1158 p*RPT6*::kan^R^-tetO_7_-TATA) were obtained from Open Biosystems. Rpt1 tet-off (R1158 p*RPT1*::kan^R^-tetO_7_-TATA), Rpt3 tet-off (R1158 p*RPT3*::kan^R^-tetO_7_-TATA), and Rpt5 tet-off (R1158 p*RPT5*::kan^R^-tetO_7_-TATA) were constructed in strain R1158 via one-step homologous replacement of native promoters with a kan^R^-TetO_7_-TATA-CYC1 cassette, according to Hughes *et al* (Fig 1C) [18, 19]. The correct integration was verified by polymerase chain reaction (PCR) across the junctions and again using gene specific primers.

### Plasmids

N-terminally HA-tagged yeast and mouse Rpt1–Rpt6 were expressed in the pAUR123 vector (Takara Bio). Three base subcomplex expression vectors, including pCOLA-1 (Flag-Rpt1, Rpt2, His_6_-Rpt3, Rpt4, Rpt5, and Rpt6), pETDuet-1 (Rpn1, Rpn2, and Rpn13), and pACYCDuet-1 (Nas2, Nas6, Hsm3, and Rpn14) were generous gifts from Dr. Andreas Martin (UC Berkeley). Constructs were generated by a combination of seamless ligation cloning extract [37], PCR, PCR-based mutagenesis, and restriction digestion. Mutagenesis primers used in this study are listed in S1 Table. All PCR-generated constructs were verified by sequencing analysis.

### Rescue assay

Expression plasmids for Rpt subunits and empty pAUR123 vector were transformed into Rpt tet-off strains by electroporation [38] or using the Frozen-EZ Yeast Transformation II Kit (Zymo Research) according to the manufacturer’s instructions. Transformed Rpt tet-off strains were incubated in YPDA medium plus 0.5 μg/ml Aureobasidin A (AbA) and 200 μg/ml G418 overnight at 30°C. The overnight cultures were adjusted to an optical density at 600 nm (OD_600_) at 0.3. Serial dilutions were spotted onto YPDA medium agar plates containing G418, AbA, and 10 μg/ml Dox.

### Western blotting for polyubiquitinated proteins

The analysis of polyubiquitinated proteins in yeast cells was performed according to a method previously described by the Walter lab [39] and Dohlman lab (http://www.med.unc.edu/~hdohlman/TCA.html) with some modifications. Briefly, yeast strains expressing wild-type and mutant Rpt subunit were grown to early log phase (OD_600_ = 0.6–0.8); then, they were grown for 3 h additionally in the presence of 20 μg/ml Dox. At that stage, collected yeast cells were rapidly frozen in liquid nitrogen and then lysed with glass beads in TCA buffer [10 mM Tris-HCl (pH 8.0), 10% TCA, 25 mM NH_4_OAc, 1 mM Na_2_EDTA]. Cell lysate was centrifuged and the TCA pellet was dissolved in a resuspension buffer (0.1 M Tris-HCl (pH 11), 3% SDS) at 98°C for 10 min. Total protein concentrations were quantified using the Bradford assay, and equal amount of total protein was loaded on SDS-PAGE. Polyubiquitinated proteins were quantified by Western blot using mouse monoclonal antibody directed against polyubiquitin chain (FK2, Wako) and IRdye800-conjugated anti-mouse IgG. Protein loading levels were estimated by western blot for PGK1 (22C5D8, MitoScience). Fluorescence intensities of the secondary antibody were estimated by direct infrared fluorescence imaging (Odyssey Fc, LICOR Biosciences).

### Tandem affinity purification of base subcomplexes

Purification of the base subcomplex was performed as previously described [12], with minor modifications. Three expression vectors for the base subcomplex were cotransformed into *E*. *coli* BL21(DE3) cells. The bacteria were grown at 37°C, and expression was induced with 1.0 mM isopropyl β-D-1-thiogalactopyranoside (IPTG) for 12–16 h at 20°C after the culture reached an optical density at 600 nm of 0.6. Harvested cells were resuspended in TAP buffer [25 mM HEPES (pH 7.6), 100 mM NaCl, 100 mM KCl, 10% glycerol, 10 mM MgCl_2_, 0.5 mM EDTA, and 1 mM ATP] supplemented with a protease inhibitors cocktail (Sigma-Aldrich) and DNase and lysed by sonication on ice. The centrifuged supernatant was applied to a His-Select Spin column (Sigma-Aldrich) equilibrated with TAP buffer. Proteins were eluted from the column with TAP buffer containing 150 mM imidazole and 5 mM 2-mercaptoethanol. The eluted fractions were bound to a 50-μl anti-FLAG M2 beads (Sigma-Aldrich), and the base subcomplexes were eluted from the beads with TAP buffer containing 200 μg/ml 3× FLAG peptide and 5 mM 2-mercaptoethanol. After separation by 10% SDS-PAGE gel, eluted base subcomplexes were either analyzed by western blotting using standard protocols or stained with Oriole stain (Bio-Rad Laboratories). In western blotting analysis, FLAG-Rpt1 and His_6_-Rpt3 were detected with a mouse monoclonal anti-DYKDDDDK-tag antibody (1E6; Wako) and rabbit anti-His—tag antibody (MBL), followed by IRDye800-labeled donkey anti-mouse secondary antibody (LI-COR) and IRDye680-labeled goat anti-rabbit secondary antibody (LI-COR), respectively. Protein amounts were estimated by direct infrared fluorescence imaging (Odyssey Fc; LI-COR).

### Bioinformatics

The algorithms Coils [40] and Paircoil2 [41] were used for CC predictions. Both programs were used for subsequent analyses. For graphic uniformity with the Coils plots, the graphs of Paircoil2 predictions in the figures display the per-residue CC propensity as 1 minus the p-score assigned to each amino acid as an estimate of CC probability.

## Supporting Information

S1 Fig Multiple sequence alignment of six yeast Rpt subunits. Sequence conservation is indicated beneath the alignment, and conserved residues are marked and color-coded according to the default ClustalX settings.
(TIF)

S2 Fig Native PAGE analysis of the proteasome assembly in Rpt tet-off strains. Assembly of the proteasome in Rpt tet-off strains in the absence and presence of Dox was analyzed by native PAGE gel. After yeast cells at early log phase were treated with 20 μg/mL Dox for 6 h, cells were harvested and lysed with glass beads. Lysed cell lysate were loaded into the native PAGE gel with loading buffer. The gel was stained for LLVY-AMC hydrolytic activity in the presence of 0.05% SDS. The 26S proteasome migrated as two bands corresponding to proteasomes core particle (CP) singly (CP-RP_1_) or doubly (CP-RP_2_) capped with regulatory particles (RP).
(TIF)

S3 Fig Expression of yeast and mouse Rpt subunits encoded in the pAUR123 vector in Rpt tet-off strains. Yeast and mouse Rpt subunits were expressed in Rpt tet-off strains in the absence of doxycycline and analyzed by western blotting. The Rpt subunits were detected using anti-HA antibody as the primary antibody and IRdye680-conjugated anti-mouse IgG as the secondary antibody. Protein loading levels in each lane were estimated in all lysates by western blotting for PGK1 (22C5D8, MitoScience).
(TIF)

S4 Fig Multiple sequence alignment of full-length Rpt subunits derived from yeast, worm, fly, frog, and mouse. The coiled-coil regions of Rpt subunits predicted by PairCoil2 are indicated above the sequences. Sequence conservation is indicated beneath the alignment, and conserved residues are marked and color-coded according to the default ClustalX settings.
(TIF)

S5 Fig The N-terminal regions of Rpt subunits are largely disordered. Prediction of intrinsically disordered regions in yeast Rpt subunits using the DISOPRED server. The N-terminal regions of all Rpt subunits are largely disordered, whereas internal OB and ATPase domains are well structured. Blue line shows disorder confidence levels against the sequence positions. Gray dashed horizontal line marks the threshold above which amino acids are regarded as disordered. Orange line shows the confidence of disordered residues being involved in protein—protein interactions.
(TIF)

S6 Fig Coiled-coil probability for yeast Rpt subunits obtained using Paircoil2 and Coils. These graphs show the coiled-coil probability over the sequence of N-terminal regions of Rpt subunits. For graphic uniformity with the Coils plots (blue lines), the graphs of Paircoil2 (red lines) predictions in the figures display the per-residue coiled-coil propensity as 1 minus the p-values assigned to each amino acid as an estimate of coiled-coil probability. The thickness of the red lines represents the p-scores predicted by Paircoil2. Coils prediction uses sliding windows of 28 (solid line), 21 (dashed line), and 14 (dotted line) residues.
(TIF)

S7 Fig Expression of a series of deletion mutant of Rpt subunits encoded in the pAUR123 vector in Rpt tet-off strains. A series of deletion mutants of Rpt subunits were expressed in Rpt tet-off strains in the absence of doxycycline and analyzed by western blotting. The Rpt subunits were detected using anti-HA antibody as the primary antibody and IRdye680-conjugated anti-mouse IgG as the secondary antibody. Protein loading levels in each lane were estimated in all lysates by western blotting for PGK1.
(TIF)

S8 Fig Expression of coiled-coil mutations of Rpt subunits encoded in the pAUR123 vector in Rpt tet-off strains. CC−, CC0, and CC00 mutants of Rpt subunits were expressed in Rpt tet-off strains in the absence of doxycycline and analyzed by western blotting. The Rpt subunits were detected using anti-HA antibody as the primary antibody and IRdye680-conjugated anti-mouse IgG as the secondary antibody. Protein loading levels in each lane were estimated in all lysates by western blotting for PGK1.
(TIF)

S9 Fig CC0 and CC00 mutants do not have a decreased coiled-coil probability. Coiled-coil probability for N-terminal region of wild-type (black lines), CC0 mutants (orange lines), and CC00 (red broken lines) mutants of Rpt subunits predicted by Paircoil2.
(TIF)

S1 Protocol Expression analysis of HA-tagged Rpt subunits. (DOCX)

S2 Protocol Native PAGE analysis of the proteasome assembly (DOCX)

S1 Table List of mutagenesis primers used in this study. (XLSX)
