# Supplementary figures and images for: N-Terminal Coiled-Coil Structure of ATPase Subunits of 26S Proteasome Is Crucial for Proteasome Function
Source: PLoS One. 2015 Jul 24;10(7):e0134056. doi: 10.1371/journal.pone.0134056 (PMC4514846; doi:10.1371/journal.pone.0134056)

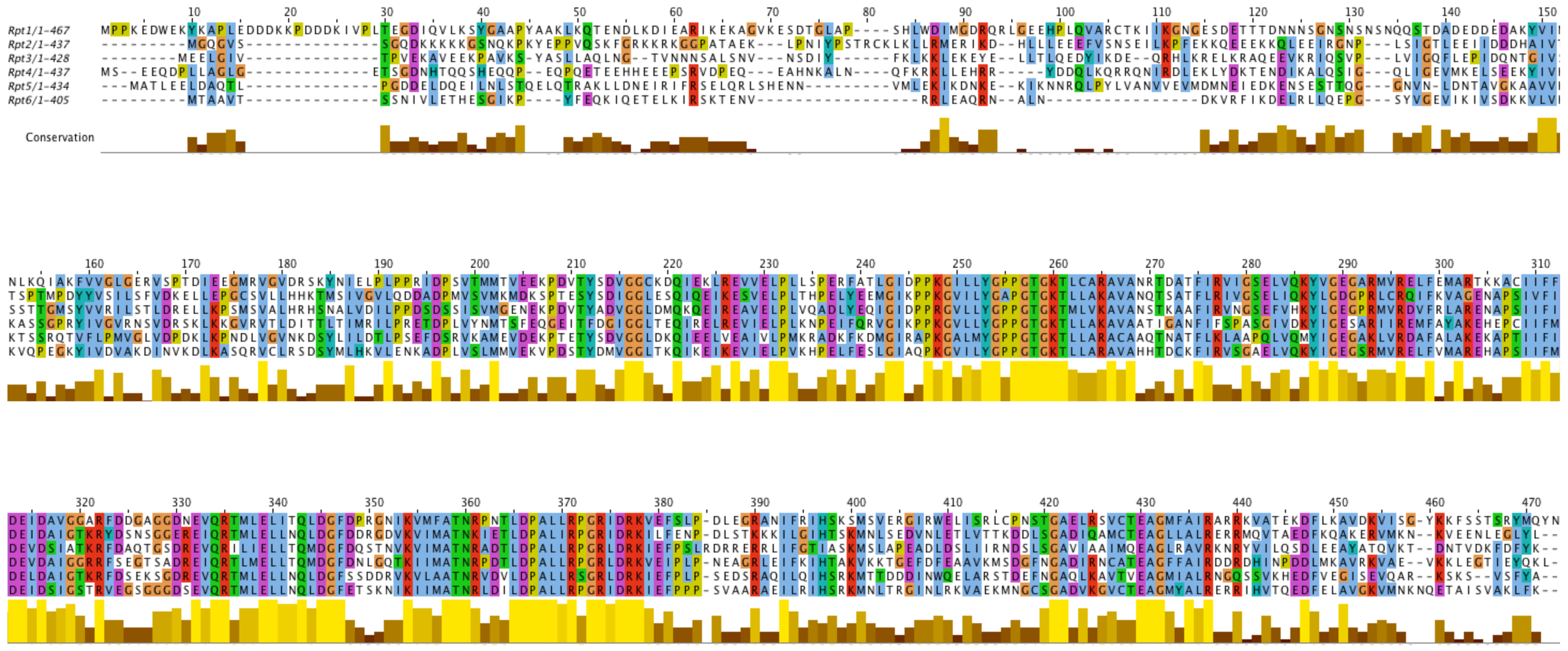

Supplement: S1 Fig — Sequence conservation is indicated beneath the alignment, and conserved residues are marked and color-coded according to the default ClustalX settings. (TIF) [file pone.0134056.s001.tif]

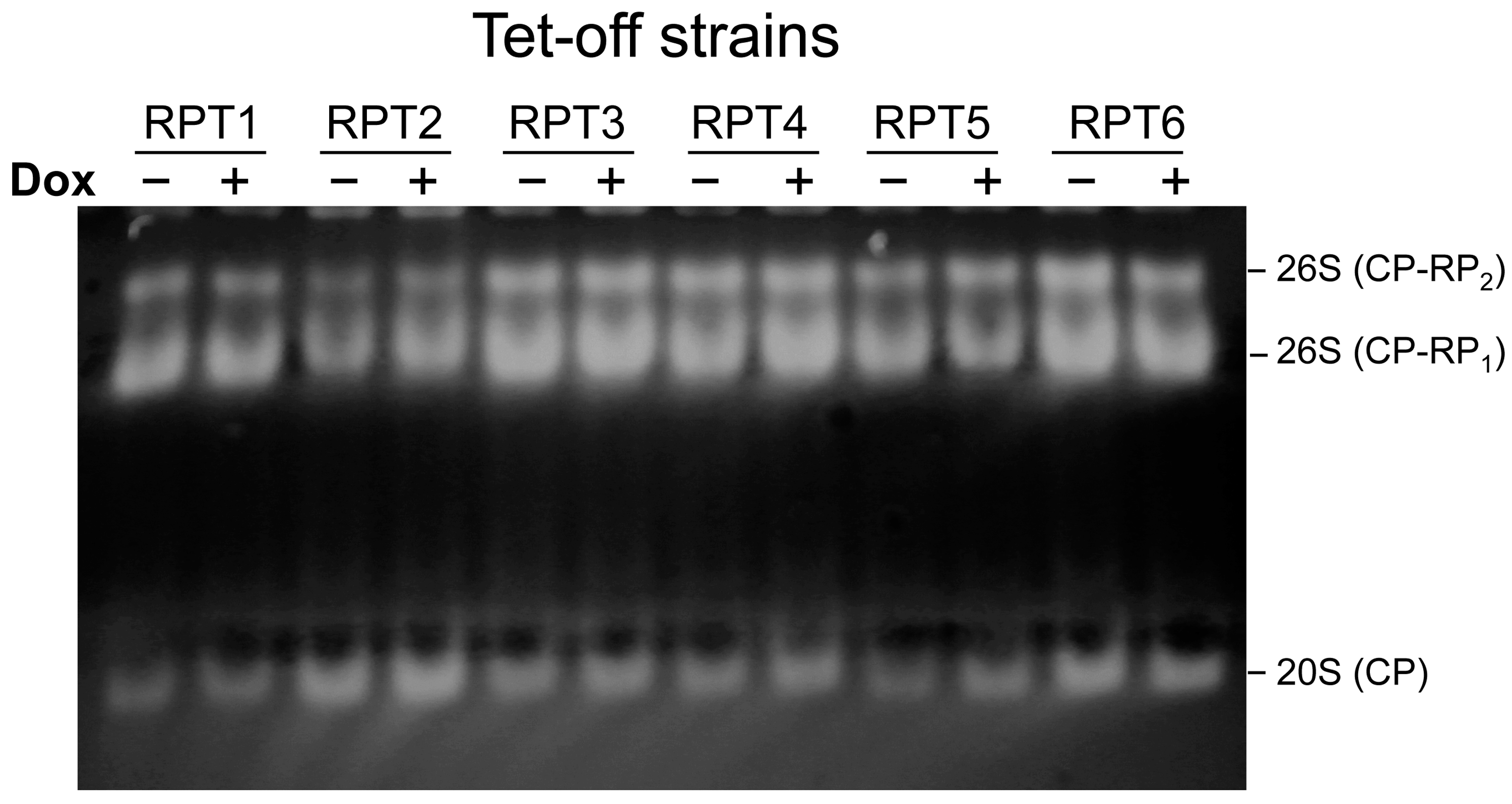

Supplement: S2 Fig — Assembly of the proteasome in Rpt tet-off strains in the absence and presence of Dox was analyzed by native PAGE gel. After yeast cells at early log phase were treated with 20 μg/mL Dox for 6 h, cells were harvested and lysed with glass beads. Lysed cell lysate were loaded into the native PAGE gel with loading buffer. The gel was stained for LLVY-AMC hydrolytic activity in the presence of 0.05% SDS. The 26S proteasome migrated as two bands corresponding to proteasomes core particle (CP) singly (CP-RP1) or doubly (CP-RP2) capped with regulatory particles (RP). (TIF) [file pone.0134056.s002.tif]

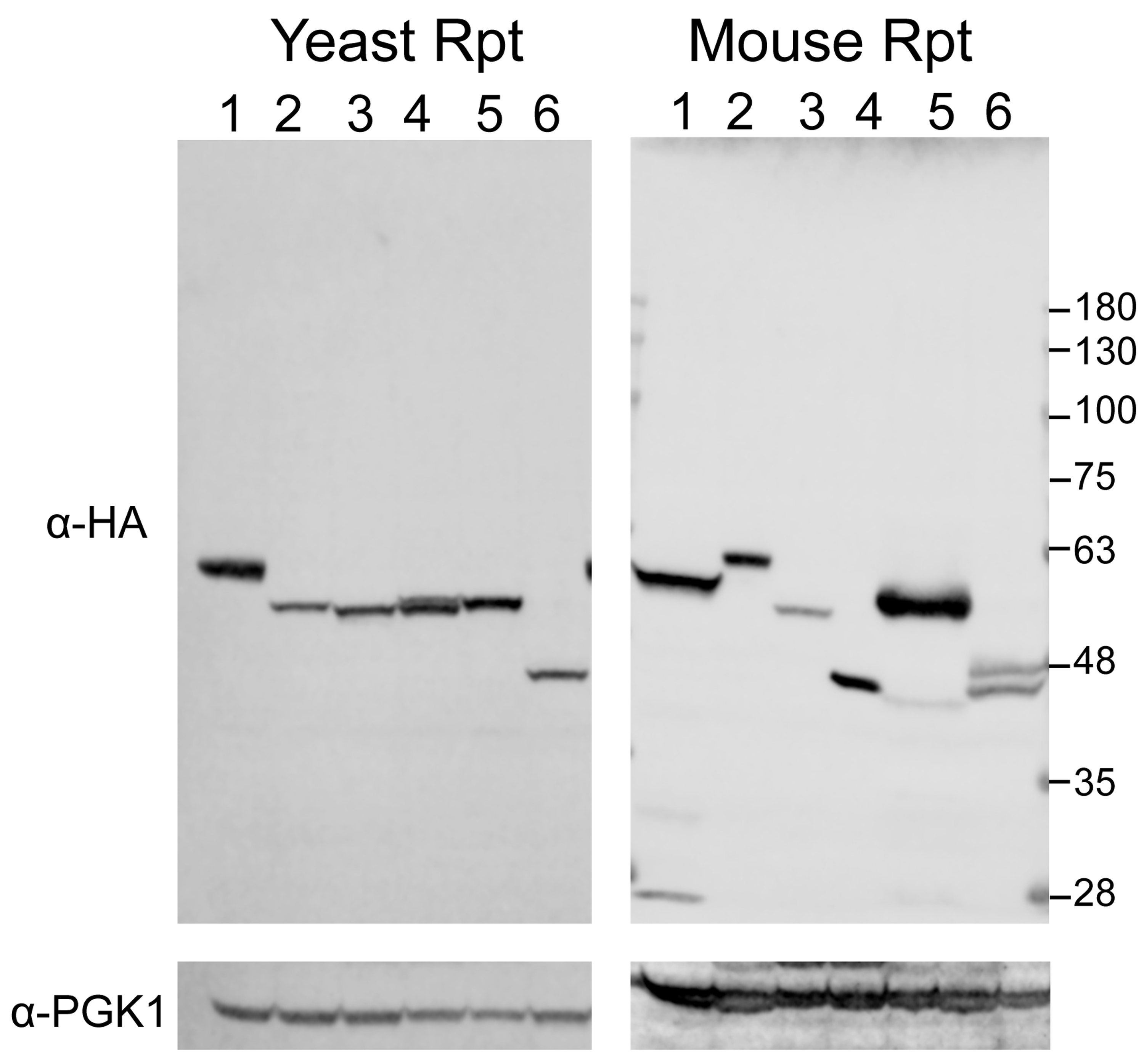

Supplement: S3 Fig — Yeast and mouse Rpt subunits were expressed in Rpt tet-off strains in the absence of doxycycline and analyzed by western blotting. The Rpt subunits were detected using anti-HA antibody as the primary antibody and IRdye680-conjugated anti-mouse IgG as the secondary antibody. Protein loading levels in each lane were estimated in all lysates by western blotting for PGK1 (22C5D8, MitoScience). (TIF) [file pone.0134056.s003.tif]

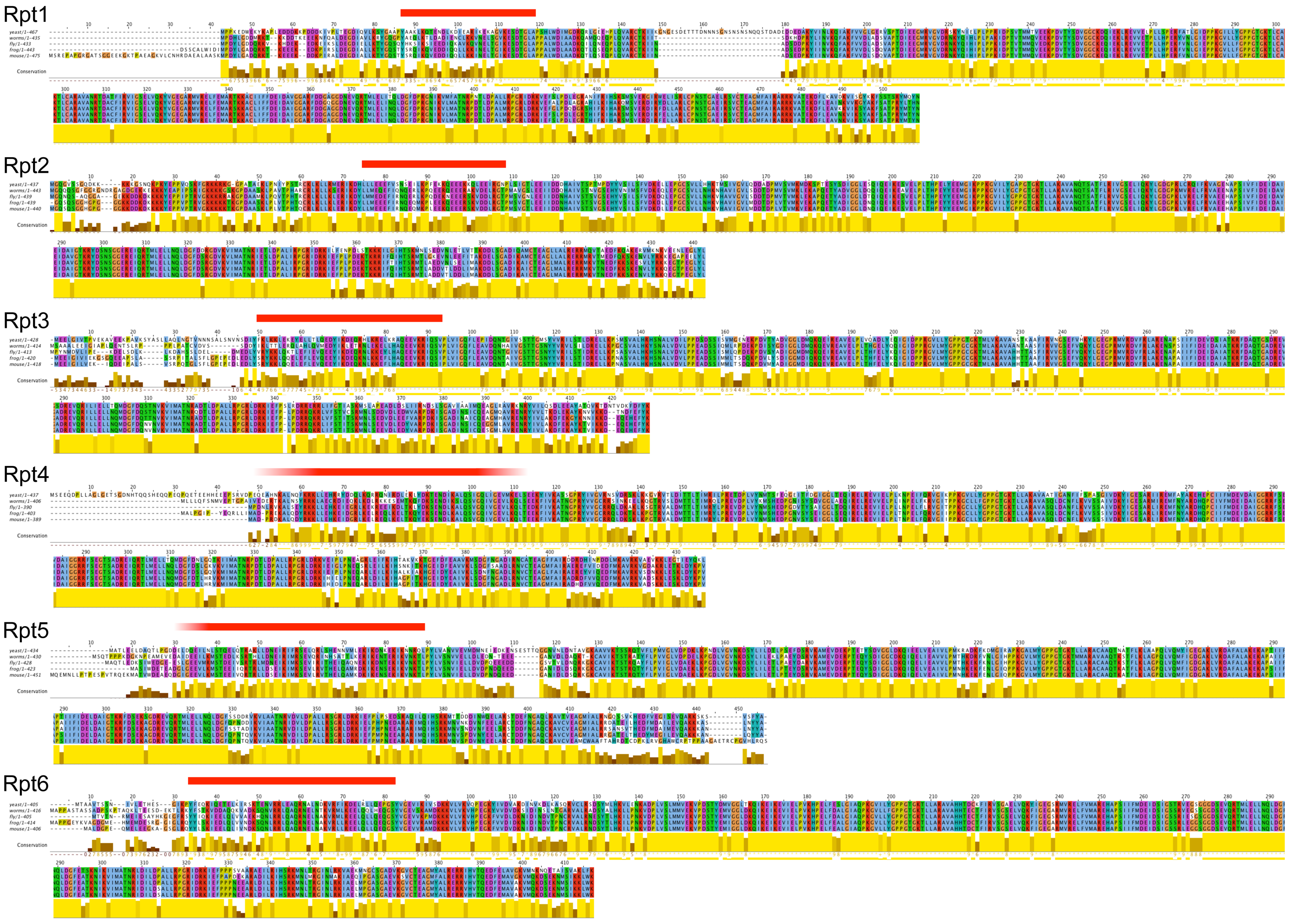

Supplement: S4 Fig — The coiled-coil regions of Rpt subunits predicted by PairCoil2 are indicated above the sequences. Sequence conservation is indicated beneath the alignment, and conserved residues are marked and color-coded according to the default ClustalX settings. (TIF) [file pone.0134056.s004.tif]

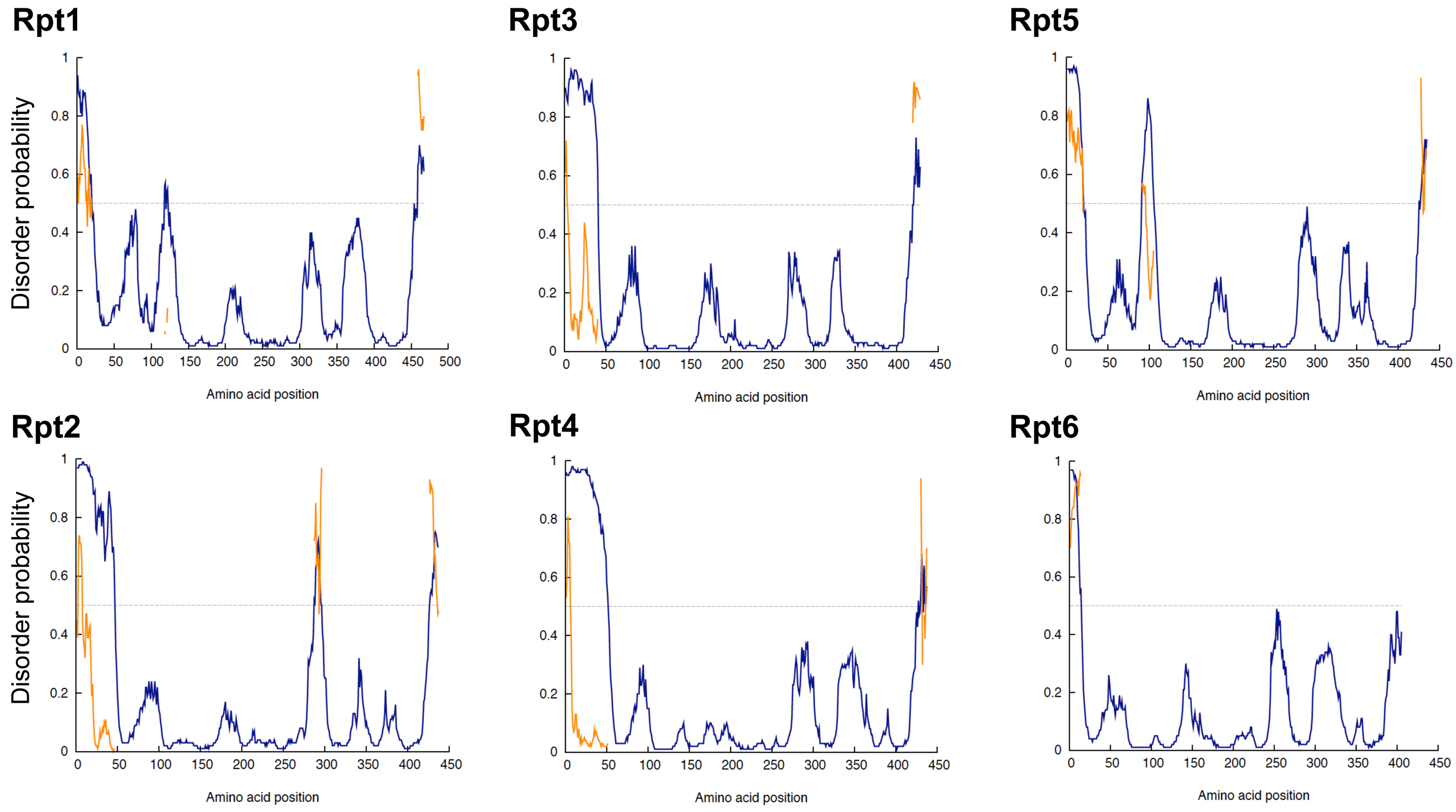

Supplement: S5 Fig — Prediction of intrinsically disordered regions in yeast Rpt subunits using the DISOPRED server. The N-terminal regions of all Rpt subunits are largely disordered, whereas internal OB and ATPase domains are well structured. Blue line shows disorder confidence levels against the sequence positions. Gray dashed horizontal line marks the threshold above which amino acids are regarded as disordered. Orange line shows the confidence of disordered residues being involved in protein—protein interactions. (TIF) [file pone.0134056.s005.tif]

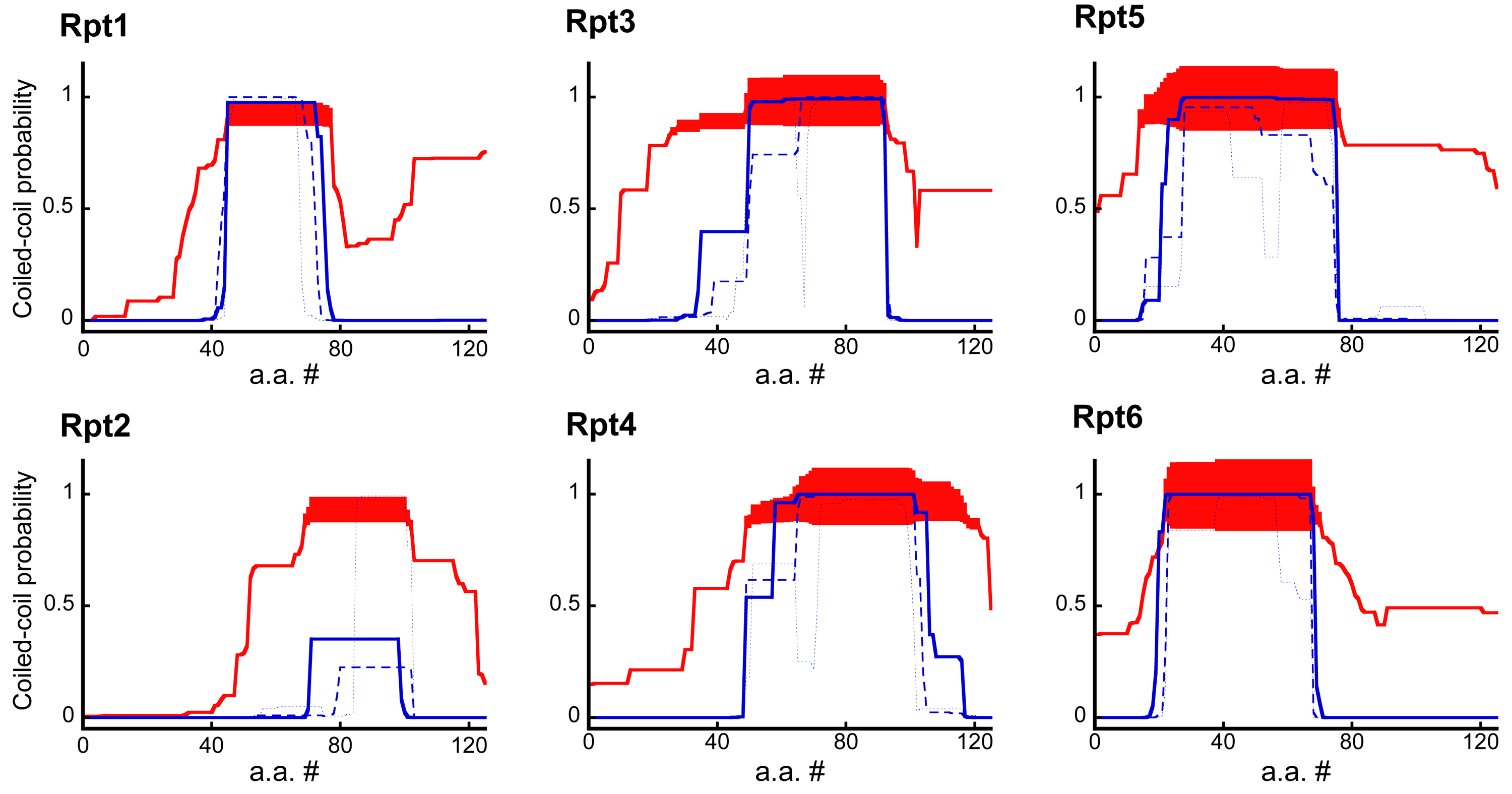

Supplement: S6 Fig — These graphs show the coiled-coil probability over the sequence of N-terminal regions of Rpt subunits. For graphic uniformity with the Coils plots (blue lines), the graphs of Paircoil2 (red lines) predictions in the figures display the per-residue coiled-coil propensity as 1 minus the p-values assigned to each amino acid as an estimate of coiled-coil probability. The thickness of the red lines represents the p-scores predicted by Paircoil2. Coils prediction uses sliding windows of 28 (solid line), 21 (dashed line), and 14 (dotted line) residues. (TIF) [file pone.0134056.s006.tif]

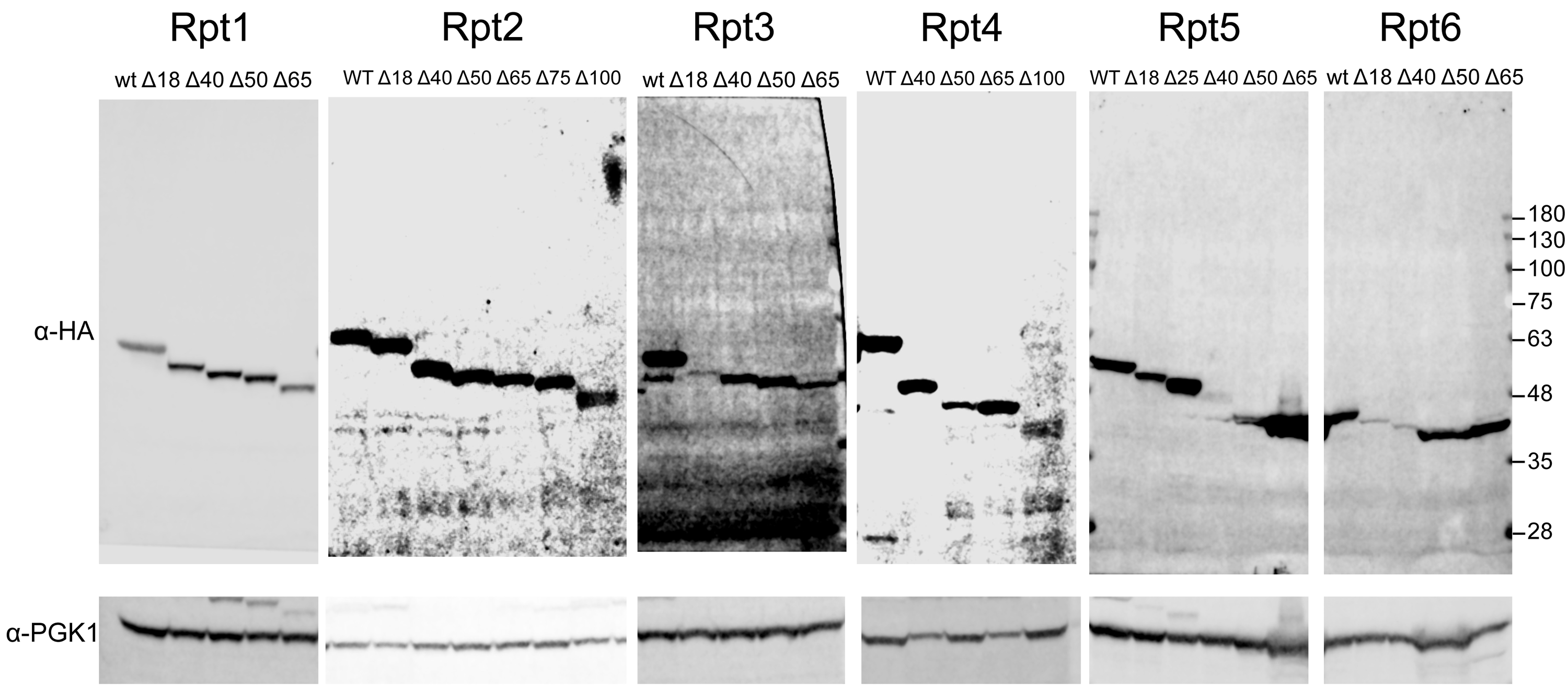

Supplement: S7 Fig — A series of deletion mutants of Rpt subunits were expressed in Rpt tet-off strains in the absence of doxycycline and analyzed by western blotting. The Rpt subunits were detected using anti-HA antibody as the primary antibody and IRdye680-conjugated anti-mouse IgG as the secondary antibody. Protein loading levels in each lane were estimated in all lysates by western blotting for PGK1. (TIF) [file pone.0134056.s007.tif]

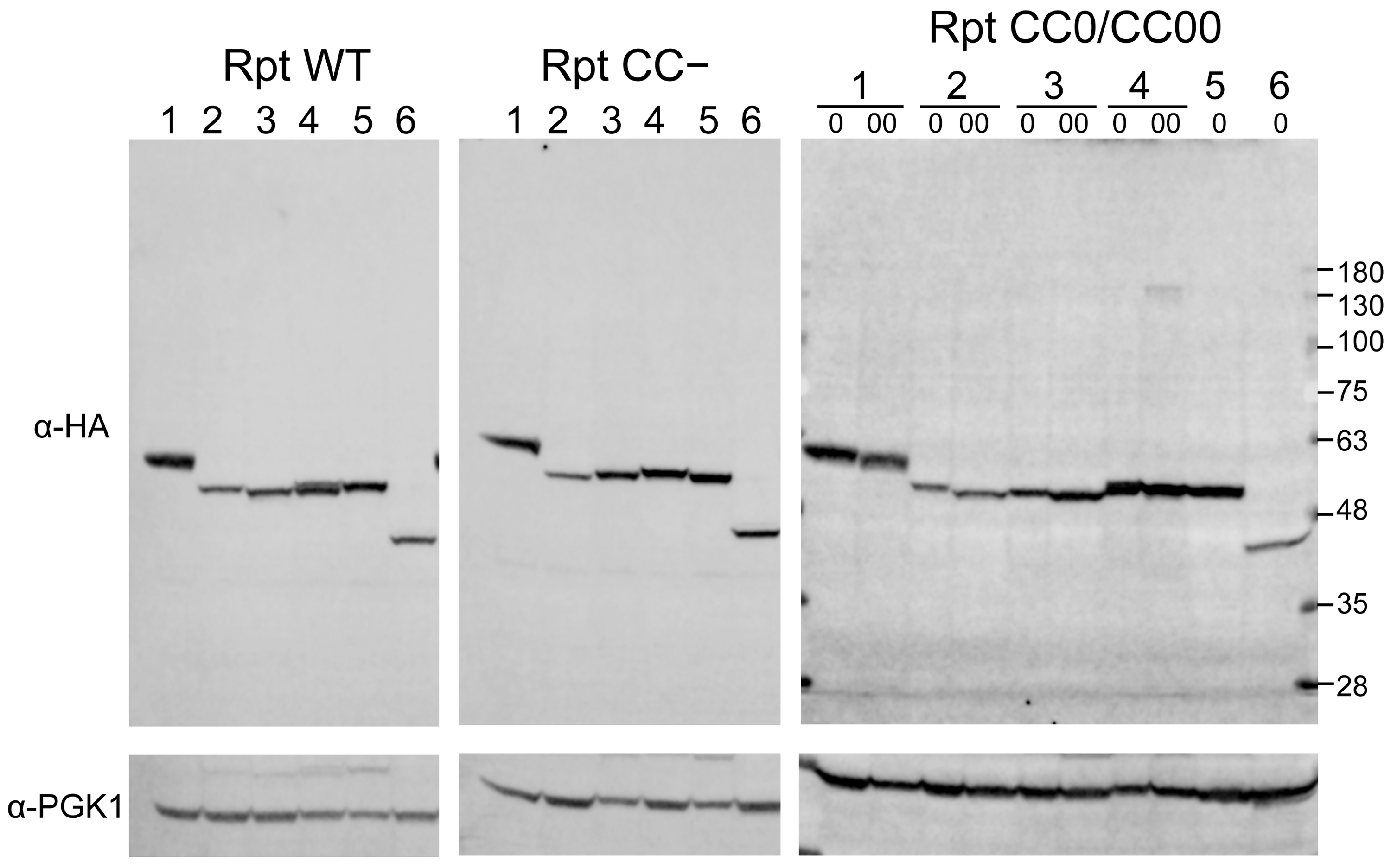

Supplement: S8 Fig — CC−, CC0, and CC00 mutants of Rpt subunits were expressed in Rpt tet-off strains in the absence of doxycycline and analyzed by western blotting. The Rpt subunits were detected using anti-HA antibody as the primary antibody and IRdye680-conjugated anti-mouse IgG as the secondary antibody. Protein loading levels in each lane were estimated in all lysates by western blotting for PGK1. (TIF) [file pone.0134056.s008.tif]

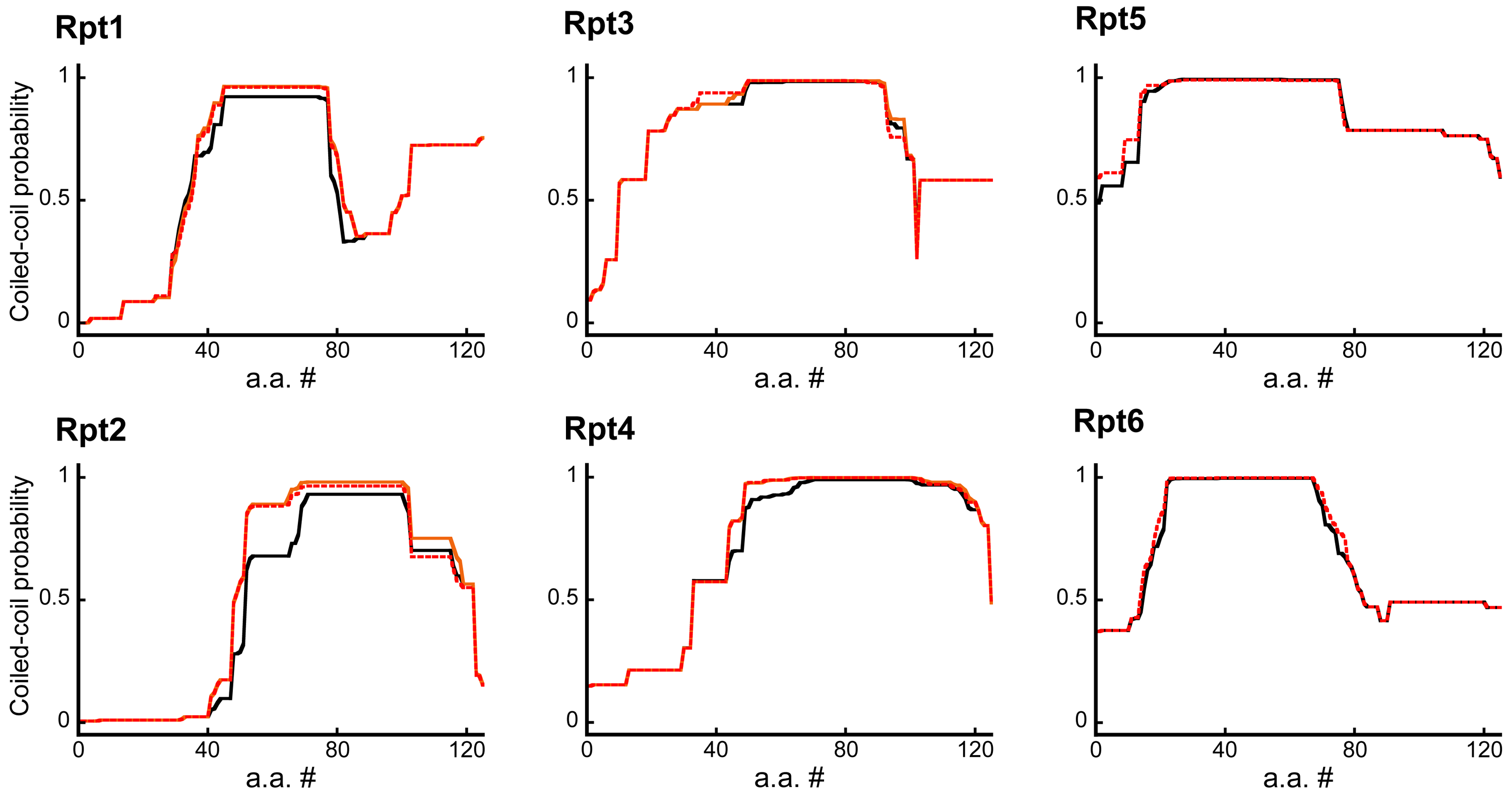

Supplement: S9 Fig — Coiled-coil probability for N-terminal region of wild-type (black lines), CC0 mutants (orange lines), and CC00 (red broken lines) mutants of Rpt subunits predicted by Paircoil2. (TIF) [file pone.0134056.s009.tif]
