# Supplementary material for: N-Terminal Coiled-Coil Structure of ATPase Subunits of 26S Proteasome Is Crucial for Proteasome Function
Source: PLoS One. 2015 Jul 24;10(7):e0134056. doi: 10.1371/journal.pone.0134056 (PMC4514846; doi:10.1371/journal.pone.0134056)
Supplement: S1 Protocol — (DOCX) [file pone.0134056.s010.docx]

**S1 Protocol**

*Expression analysis of HA-tagged Rpt subunits*

To check the expression of HA-tagged Rpt subunits, total protein was extract from yeast as described by Zhang et al. Briefly, Yeast cells grown in YPDA medium plus 0.5 μg/ml Aureobasidin A (AbA) and 200 μg/ml G418 at 30°C were harvested when OD_600_ reached 1.0. Cells were first resuspended in 0.5 ml of 2 M lithium acetate for 5 min on ice. After centrifugation at 5000 ×g for 1min, cells were resuspended in 0.5 ml of 0.4 M NaOH for 5 min on ice. The cells were centrifuged again at 5000 ×g for 1 min and then resuspended in 200 µl of SDS sample buffer [50 mM Tris-HCl (pH6.8), 10% Glycerol, 2% SDS, 0.05%(w/v) Orange G] and heated at 95 °C for 5 min. Cell debris was spun down at 22,000 × g for 10 min, and the supernatants were collected. The total proteins in the supernatants were separated on a 10% SDS-PAGE gel and transfered to immobilon-FL PVDF membrane (Merck Millipore). HA-tagged Rpt subunits were detected with mouse anti-HA monochronal antibody (TANA2; MBL) and IRDye680-labeled goat anti-mouse secondary antibody (LI-COR), using an infrared imaging system (Odyssey Fc; LI-COR).
