# Supplementary material for: N-Terminal Coiled-Coil Structure of ATPase Subunits of 26S Proteasome Is Crucial for Proteasome Function
Source: PLoS One. 2015 Jul 24;10(7):e0134056. doi: 10.1371/journal.pone.0134056 (PMC4514846; doi:10.1371/journal.pone.0134056)
Supplement: S2 Protocol — (DOCX) [file pone.0134056.s011.docx]

**S2 Protocol**

*Native PAGE analysis of the proteasome assembly*

Analysis of proteasome assembly in yeast cell extracts by native gels was performed as described previously (Elsasser *et al*, Methods in Enzymology vol. 398, 353-363). Briefly, 4% polyacrylamide native gel was made based on Tris-borate buffer (90 mM Tris base, 90 mM boric acid, 0.1 mM EDTA, pH 8.35). Rpt tet-off strains were grown to early log phase (OD_600_ = 0.6-0.8) and then grown for six more hours in the presence 20 μg/ml Dox. Cells were harvested and lysed with glass beads in lysis buffer (50 mM Tris-HCl (pH 7.5), 5 mM MgCl_2_, 4 mM ATP, 50 mM NaCl, 10% glycerol)). Lysed cell lysate were mixed with 6X loading buffer (300 mM Tris-HCl (pH 7.5), 60% glycerol) just before loading. Electrophoresis was carried out in Tris-borate buffer containing 1mM ATP and 5mM MgCl_2_ at 4°C and 100V for 4h. The gel was overlaid with developer solution (running buffer with 100 nM Suc-LLVY-AMC peptide and 0.02% SDS) and incubated at 30 °C for 10 min before imaging. Fluorescence image of the gel was photographed on the UV transilluminator.
